# Anthropogenic climate change has driven over 5 million km^2^ of drylands towards desertification

**DOI:** 10.1038/s41467-020-17710-7

**Published:** 2020-07-31

**Authors:** A. L. Burrell, J. P. Evans, M. G. De Kauwe

**Affiliations:** 1 Woods Hole Research Center, 149 Woods Hole Road, Falmouth, MA 02540 USA; 2 Centre for Landscape and Climate Research, School of Geography, Geology and the Environment, University of Leicester, University Road, Leicester, LE1 7RH UK; 3 Climate Change Research Centre, University of New South Wales, Sydney, NSW 2052 Australia; 4 ARC Centre of Excellence for Climate Extremes, University of New South Wales, Sydney, NSW 2052 Australia; 5 Evolution and Ecology Research Centre, University of New South Wales, Sydney, NSW 2052 Australia

**Keywords:** Attribution, Environmental health

## Abstract

Drylands cover 41% of the earth’s land surface and include 45% of the world’s agricultural land. These regions are among the most vulnerable ecosystems to anthropogenic climate and land use change and are under threat of desertification. Understanding the roles of anthropogenic climate change, which includes the CO_2_ fertilization effect, and land use in driving desertification is essential for effective policy responses but remains poorly quantified with methodological differences resulting in large variations in attribution. Here, we perform the first observation-based attribution study of desertification that accounts for climate change, climate variability, CO_2_ fertilization as well as both the gradual and rapid ecosystem changes caused by land use. We found that, between 1982 and 2015, 6% of the world’s drylands underwent desertification driven by unsustainable land use practices compounded by anthropogenic climate change. Despite an average global greening, anthropogenic climate change has degraded 12.6% (5.43 million km^2^) of drylands, contributing to desertification and affecting 213 million people, 93% of who live in developing economies.

## Abstract (web summary)

Drylands cover nearly half of Earth’s surface, yet how they will fare in light of anthropogenic climate change is debated. Here the authors find that over the past 40 years climate change has pushed ~13% of drylands towards desertification threatening hundreds of millions of people in developing nations.

## Introduction

Land degradation is a systemic global problem^1–4^ but the scale of the problem is disputed, with global estimates of degraded areas ranging from <10 to >60 million km^2^ ^5^. Changes in vegetation in drylands are predominantly caused by two factors: (i) anthropogenic climate change (ACC), which includes both changes in water availability driven by trends in precipitation and increases in temperature^6,7^, as well as increased water use efficiency (carbon gain per unit of water lost) in response to rising atmospheric CO_2_^8^; and (ii) land use (LU) practices, including grazing, cropping and deforestation^2,9^. Unsustainable LU is considered the primary negative driver of dryland degradation^9–11^. The impact of climate change (CC) on drylands is also generally thought to be negative, with some studies suggesting that anthropogenic forcing has already increased arid areas^12–14^.

Despite evidence for LU-induced degradation and the studies that find increased aridification over drylands, satellite estimates of vegetation greenness (a proxy for net primary productivity (NPP)) show a significant global increase since 1980^10^. The key drivers of this global increase in apparent vegetation productivity are the vegetation’s response to rising CO_2_^8,15^, increases in rainfall and temperature^16,17^ and LU^10^. Model simulations which prescribe LU, attribute almost all of the trend in satellite-derived greening to CO_2_ fertilization^15^, while satellite-derived models that do not account for CO_2_, explicitly find either climate or LU as the dominate factor^10,17^. Neither approach explicitly accounts for rapid ecosystem change (break points) in their proportioning of the relative contributions of each driver. This can lead them to miss or underestimate rapid changes driven by processes like extreme fires, deforestation, reforestation, changes in agricultural policy, etc.^18–21^. Disentangling the roles of climate (temperature and precipitation), CO_2_ and LU thus remains a key challenge^22^ and has been identified as a key knowledge gap by the United Nations Convention to Combat Desertification^2^ (UNCCD), the Intergovernmental Panel on Climate Change^23^ (IPCC), and the Intergovernmental Science–Policy Platform on Biodiversity and Ecosystem Services^3^.

Here we quantified the scale of global desertification, which both the UNCCD and IPCC define as degradation in arid, semi-arid, and dry sub-humid areas^23^. These definitions further define degradation as the long-term reduction or loss of biological productivity among other things. Here we identify areas undergoing long-term reductions in vegetation in dryland areas, hence the desertification according to relevant international conventions, using the satellite-based GIMMSv3.1g Normalized Difference Vegetation Index (NDVI) data. We calculated the overall vegetation change using a non-parametric trend analysis applied to peak growing season NDVI (NDVI_max_). We then attributed this change to CO_2_, climate variability (CV), CC, and LU using a modified version of the Time Series Segmented Residual Trends (TSS-RESTREND) method^19,24^. This approach quantifies the effect of interannual CV as well as long-term changes in climate and CO_2_ fertilization in addition to ecosystem break points caused by LU (see “Methods”). To quantify uncertainties, we used a 12-member ensemble made up of statistical model runs performed using a combination of observation-based gridded datasets (four precipitation and three temperature datasets). We show that 6% of dryland areas have undergone desertification since 1982 with a further 20% of dryland areas being at high risk of future desertification as a result of unsustainable LU practices and ACC.

## Results and discussion

### The extent and drivers of dryland vegetation change

**Fig. 1:**
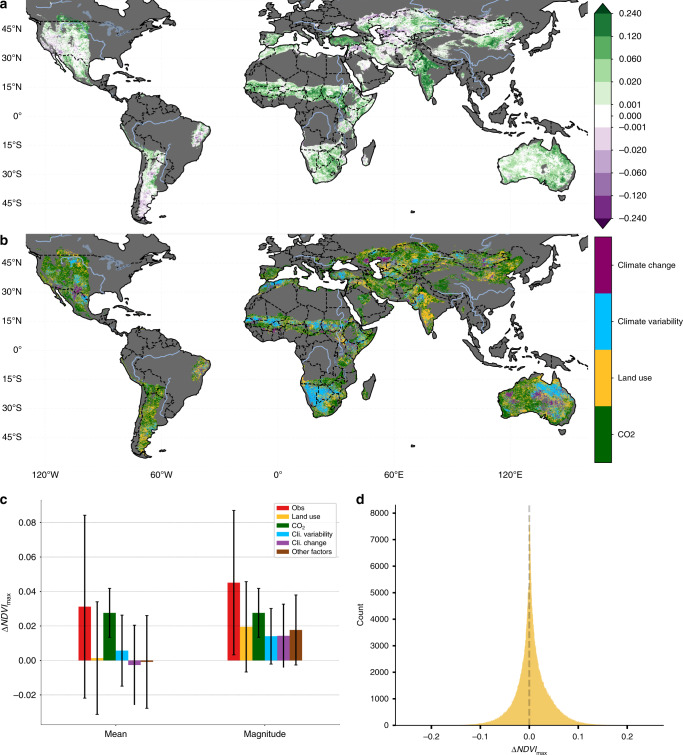
Primary drivers of vegetation changes between 1982 and 2015. **a** The observed dryland vegetation change from 1982 to 2015 measured using the change in *p* Normalized Difference Vegetation Index (ΔNDVI_max_). Non-dryland and hyper-arid regions are masked in dark gray and areas where the change is insignificant (*α*_FDR_ = 0.10) or smaller than the error in the sensors (±0.001) are masked in white. **b** Map showing the largest absolute attributed driver (CO_2_, land use, climate change, and climate variability). Non-dryland regions are masked in dark gray and the magnitude of other factors is not considered. **c** The mean and magnitude (mean absolute value) of the different drivers of change in NDVI_max_ between 1982 and 2015. The error bars show the SD of the area-weight grid cells. **d** Frequency distribution function for the change in NDVI_max_ (1982–2015) attributed to land use, which shows that there are opposing changes at local scales which cancel out when averaged globally.

Globally, of the 44.5 million km^2^ of drylands, 6% of these areas experienced desertification (i.e., significant negative change in NDVI_max_), 41% showed significant greening (i.e., significant positive change), and 53% had no significant change between 1982 and 2015 (Fig. 1a). The mean (±1 SD) of the area-weighted dryland vegetation change, as represented by the change in NDVI_max_ was 0.031 ± 0.053. We estimated the scale of desertification to be 2.70 million km^2^, which is significantly below a previous estimate of ~10.5 million km^2^ over the same region, but over a different time window (1982 and 2003)^1^. A large part of this discrepancy can be attributed to climatic differences in the end dates of the studies (2003 vs. 2015), with increased rainfall over regions including the Sahel and India^25,26^. This large difference between our estimate and this existing dryland degradation estimate highlights that time-series vegetation trend analysis is sensitive to the start and end conditions^17,18^. For this reason, understanding what is driving the observed vegetation change is more important than the current directions of vegetation change for projecting future changes and vulnerabilities. It should also be noted that although the amount of land we estimate to have experienced desertification has decreased by ~70% between these two estimates, the number of people impacted has only decreased by ~25% (250 million to 189 million)^27^.

Figure 1b shows that globally, CO_2_ fertilization was the largest absolute attributed driver of dryland vegetation change in 44.1% of areas, followed by LU (28.2%), CV (14.6%), and then CC (13.1%). However, when averaged globally (Fig. 1c), the per-pixel contribution of CO_2_ (0.021 ± 0.011) was much larger than the contribution from CV (0.006 ± 0.020), CC (−0.002 ± 0.023), or LU (0.005 ± 0.032). The relative contribution (67.8% CO_2_, −5.6% climate, 15.5% LU) fall within the range of global estimates calculated using a model based factor analysis (70.1 ± 29.4% CO_2_ fertilization, 8.1 ± 20.6% climate, 3.7 ± 14.7% LU)^15^, despite a difference in the study domains. It should be noted here that model based factor analysis did not quantify the role of CV^15^, which we find accounts for 19.4% of the observed global dryland greening between 1982 and 2015.

If only the global mean effect size is considered, climate and LU seem to have a very small impact compared with CO_2_ fertilization, which seemingly contradicts well-documented evidence of LU and climate impacts^9,11,16,17,28^, and the spatial patterns shown in Fig. 1b. For example, a recent satellite-based study, which did not consider the role of CO_2_, attributed 60% of observed global land changes to LU activities and the remaining 40% to other factors including climate^10^. For comparison, we repeated our ensemble analysis, removing the role of CO_2_ fertilization (Supplementary Fig. 1), and found that 60.4% of global dryland vegetation change would have been attributed to LU and 39.6% to CC and variability combined, which is consistent despite a difference in the study domains and the attribution methods (see Supplementary Text 1). This result underlines the need to explicitly consider the positive role of CO_2_ as a driving mechanism for change in dryland ecosystems^8^. When CO_2_ is included in the analysis, the mean effect of LU and climate are small, because there are roughly equal areas of positive and negative change that largely cancel out when averaged globally (Fig. 1c, d), a result which holds even if we assume a different level of vegetation response to elevated CO_2_ (Supplementary Fig. 2). This means it is also important to consider the magnitude of the different drivers (Fig. 1c).

### The impact of anthropogenic climate change

**Fig. 2:**
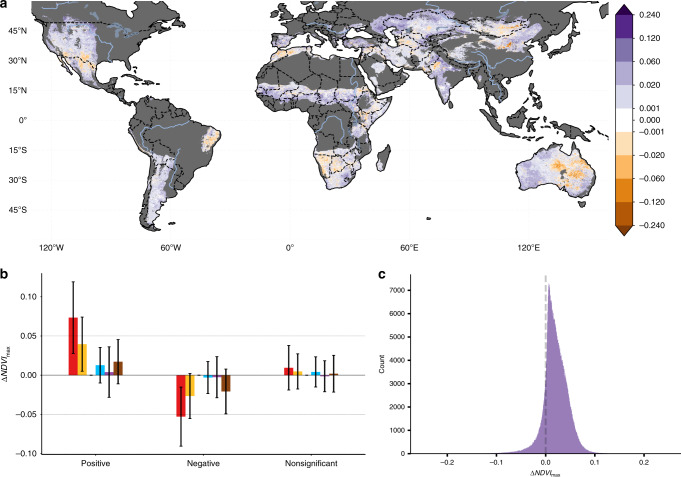
The contribution of anthropogenic climate change to vegetation change from 1982 to 2015. **a** The contribution of anthropogenic climate change (Climate Change + O_2_) component to the change in vegetation between 1982 and 2015 (ΔNDVI_max_). Non-dryland and hyper-arid regions are masked in dark gray and areas where the change is insignificant (*α*_FDR_  = 0.10) or smaller than the error in the sensors (±0.001) are masked in white. **b** The mean area-weighted pixel effect size of the drivers of observed vegetation broken down by observed vegetation change direction. Positive indicates greening and negative is desertification. Error bars show the SD. **c** Frequency distribution function for the change in NDVI_max_ (1982–2015) attributed to by anthropogenic climate change.

**Fig. 3:**
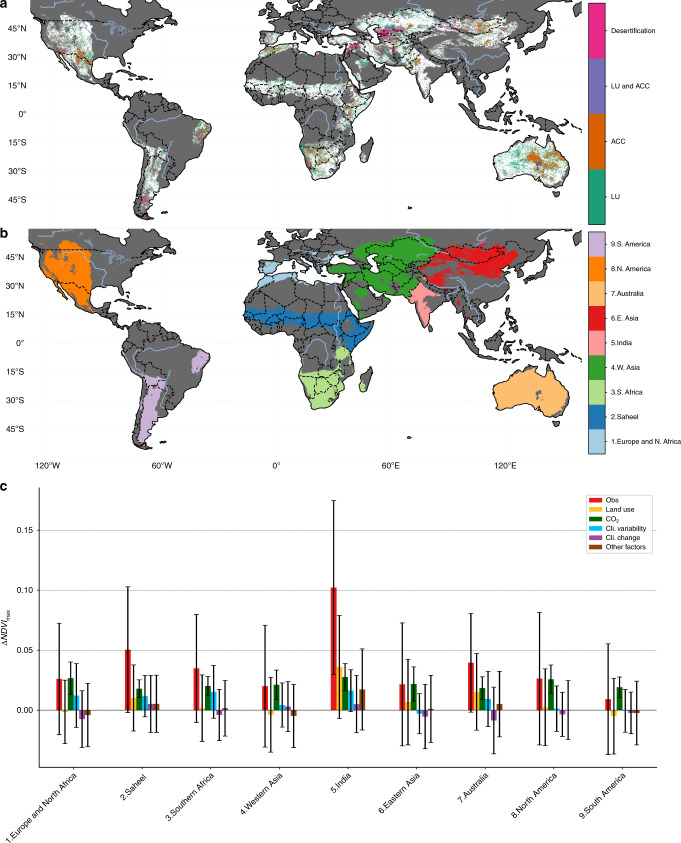
Desertification risk and regional drivers. **a** Regions experiencing, or at risk of experiencing, desertification. Areas with a significant negative change in vegetation (*α*_FDR_ = 0.10) are classified “Desertification,” areas where the anthropogenic climate change (ACC: CO_2_  + Climate Change) and land-use (LU) components are both negative but the change in vegetation is not significant are classified as “LU and ACC.” Areas where the anthropogenic climate change has had a negative effect are classified as “ACC” and areas where land use had a negative impact classified as “LU.” **b** map of the regional subdivisions used. **c** The mean per-pixel ΔNDVI_max_ and its drivers broken down by regions shown in **b**. The error bars show the SD.

Combining the change due to CO_2_ fertilization and CC provides a quantification of the role of ACC in recent desertification (Fig. 2a). Globally, we found that ACC had a positive (greening effect) over the study period (NDVI_max_: 0.019 ± 0.027). Although broadly positive, ACC also had a desertifying effect across 12.55% (5.43 million km^2^) of drylands areas. Hotspots where ACC had a desertifying effect include parts of the western United States, eastern Brazil, Iraq, Syria, Jordan, Kazakhstan, Uzbekistan, Mongolia, and Australia. Crucially, the negative effects of ACC are disproportionately felt by poorer nations with 85% of the 213.4 million people impacted living in developing or newly industrialized countries. However, a negative ACC forcing does not guarantee an area experienced desertification (Fig. 3a). Only 13.8% (0.75 million km^2^) of areas with a negative ACC forcing, experienced significant desertification (*α*_FDR_ = 0.10) and in only 2.27% (0.015 million km^2^) of the areas experiencing desertification, did we find that climate was the sole negative driver.

### Drivers of desertification

**Fig. 4:**
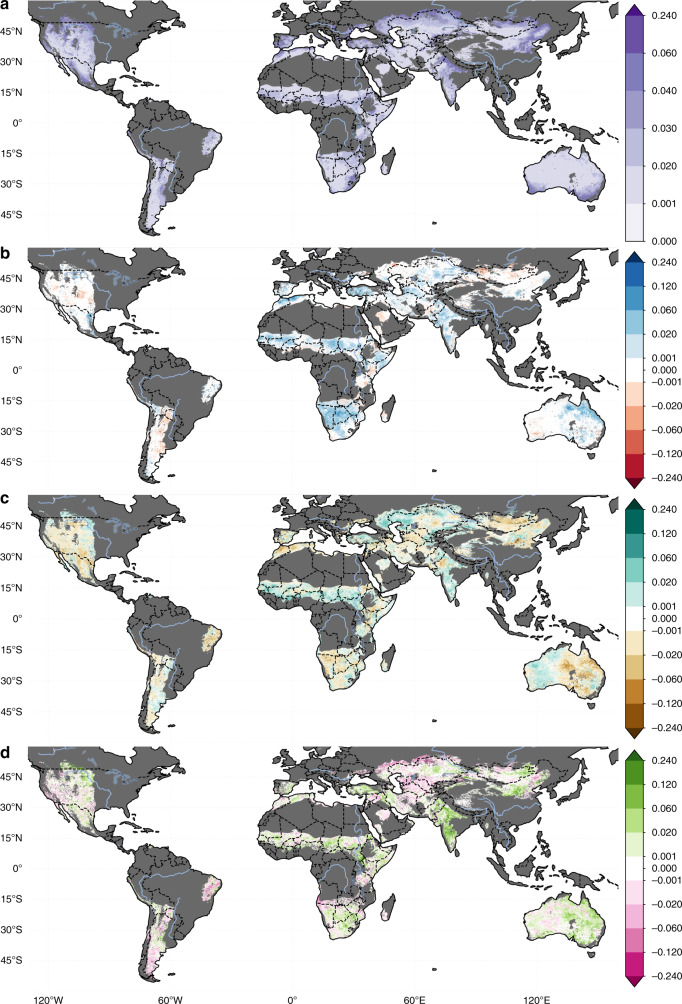
The drivers of global vegetation change. The changes in NDVI_max_ between 1982 and 2015 (ΔNDVI_max_) attributed to (**a**) CO_2_ fertilization, (**b**) climate variability, (**c**) climate change, and (**d**) land use. Non-dryland and hyper-arid regions are masked in dark gray. Areas where the change did not meet the multi-run ensemble significance criteria detailed in the “ Methods,” or are smaller than the error in the sensors (±0.001) are masked in white.

In the 2.70 million km^2^ of drylands that experienced desertification, a negative LU component was the primary driver in 79.9% and a contributing factor across 99.0% of areas (Fig. 2b). Even though the average impact of CC (NDVI_max_: −0.004 ± 0.030) and CV (−0.002 ± 0.024) are much smaller than LU (NDVI_max_: −0.040 ± 0.034), climate remains an important driver of desertification. Ecosystems that are experiencing reduced water availability or drought conditions are much more vulnerable to degradation from LU and vice versa, with the negative effects compounding^7^. For example, over parts of Central Asia we observed negative changes in both CC and LU (Fig. 4), which is consistent with the strong evidence of long-term degradation driven by unsustainable LU practices resulting in the well-documented Aral Sea disaster^26^. Similarly, the negative impacts of decreased rainfall over the semi-arid Caatinga forest of Brazil has amplified the effects of widespread deforestation and grazing intensification. The decrease in rainfall in South America results from both CC (mean precipitation anomaly 1982–2015 ≈ −0.2) as well as a negative phase of CV (mean precipitation anomaly ≤ 0 and mean temperature anomaly ≥ 0) from 2009 to 2014 (Supplementary Figs. 3 and 4). For further discussion of these regions and comparison with regional studies, see Supplementary Text 2.1. In addition to the drylands experiencing desertification, there are additional 12.0 million km^2^ and 507 million people, living in areas where the desertifying effect of LU has been offset by a positive ACC signal (Fig. 3). These regions, along with the 7.2% of areas with a negative CC, but no significant vegetation change, are at the highest risk of future desertification.

### Drivers of dryland greening

We also observed widespread global greening, with 18.0 million km^2^ of drylands having a significant positive vegetation change (Fig. 1a). CO_2_ was the largest driver of this change (Figs. 1b and 2b), in line with previous findings of a dominant CO_2_ fertilization effect on global vegetation greening^8,15^. Where our results differ from previous findings is in highlighting the importance of LU and CV. Unlike model based approaches which prescribe LU^15^ and have large variability in the simulated response of vegetation to climate^29^, our approach empirically determines the impact of climate and LU on a per-pixel basis using observations. In the regions that experienced greening, we find that CO_2_ was the largest attributed driver in ~40% of areas followed closely by LU (~38%), CV (~13%), and CC (~8%). The importance of CV and LU is especially apparent when considering regional drivers, with one or both playing a large role in the observed greening in the Sahel, India, China and Australia (Figs. 3c and 4, and Supplementary Discussion 2.2).

We used an ensemble approach to minimize the uncertainty caused by observational datasets and structural change detection to account for the ecosystem break points driven by processes like deforestation observed in ~20% of areas (Supplementary Fig. 5). Our estimate of the total attributable change (CO_2_ + LU + CC + CV) varied from the observed vegetation change by only 3% and when mapped spatially, the observed vegetation change and the total attributable change show very consistent patterns of greening and browning (Supplementary Fig. 6). Furthermore, our greening attribution results are consistent with regional studies done in the Sahel, India, China, and Australia (Supplementary Discussion 2.2).

In summary, understanding the causes of dryland degradation is an important and necessary step in targeting mitigating action that can reduce the impact of CC and prevent widespread desertification. Our change detection and attribution approach highlights the importance of accounting for the role of CO_2_ and accurately quantifying the impact of LU when considering potential drivers of change in dryland ecosystems. Our results show that, despite widespread vegetation greening, 6% of areas that have undergone desertification mostly over western Asia and South America. This desertification directly effects 190 million people. In addition, we showed that unsustainable LU practices or ACC has placed 20% of drylands at high risk of desertification. This impacts 580 million people with the risk experienced disproportionately by low socioeconomic countries. Overall, our results highlight the importance of understanding what is driving the vegetation change for projecting impacts, because without this understanding there is a high risk that mitigation strategies will fail to prevent desertification.

## Methods

### Quantifying desertification

There is no universally agreed upon definition of desertification^5,23,30^. Here we use the UNCCD definition of land degradation, which is a reduction or loss of the biological or economic productivity resulting from various factors, including climatic variations and human activities, with desertification being any land degradation in dryland ecosystems^2^. Drylands are defined by the UNCCD to be areas with an Aridity index < 0.05 or >0.65^2^. Historically, this has been measured using a linear trend applied to a satellite-derived vegetation proxy^1^. In this study, we used the growing season maximum NNDVI (NDVI_max_) as a proxy of vegetation growth. For most regions, peak growing season NDVI was determined using the maximum value in a calendar year. However, pixels where peak the occurred in December, the January and February NDVI values of the subsequent year are considered part of the previous year’s growing season.

NDVI_max_ has been found to have a highly significant correlation with NPP in a large range of different dryland ecosystem^31–33^. The Desertification chapter of the 2019 IPCC report on CC and LU both, the UNCCD definition of desertification, and NDVI_max_ as a proxy of vegetation growth^23^. It should be noted that the UNCCD definition of desertification and the trend in vegetation data used to measure it will not identify processes such as shrub encroachment, over intensification of agriculture, or the invasions by non-native species, which have been linked to degradation but can cause increases in proxies such as NDVI^23,30,34^.

To produce a comparable estimate of desertification, we used a per-pixel non-parametric trend method (Theil–Sen slope estimator and Spearman’s *ρ* significance test), applied to the satellite-derived GIMMSv3.1g NDVI_max_ dataset^35^. The global dryland vegetation change (Obs) was calculated as the difference between the expected values (E) at the start (1982) and the end (2015) of the time series $({\mathrm{{Obs}}} = E_{2015} - E_{1982})$. It should be noted that this is identical to multiplying the annual trend by the length of the time series, in this case 34 years. We report all variables using the difference between expected values at the start and end of the time series (ΔNDVI_max_) rather than an as an annual trend to account for the ecosystem break points that are detected in some locations in the middle of the time series.

This study used version 3.1 of the 1/12° Global Inventory for Mapping and Modeling Studies (GIMMS)^35^ NDVI dataset, which spans 1982–2015. Although the shorter temporal but higher spatial resolution datasets from newer sensors such Moderate Resolution Imaging Spectroradiometer (MODIS) do offer advantages, the shorter temporal record poses a serious issue in dryland ecosystems. The natural variability in dryland ecosystems is greatly impacted by decadal climate modes the most significant of which is El Niño Southern Oscillation (ENSO)^36,37^. The intensity of ENSO events varies significantly and since 1980 there have been three extreme El Niño (1982, 1997, and 2015) events that significantly impacted dryland regions around the world^38^. Even with its almost 20-year record, MODIS has only captured one of these events (2015) compared with the three present in GIMMS record. It is for this reason that GIMMS remains the most widely used dataset for vegetation trend detection and attribution studies^15^.

### Accounting for the CO_2_ fertilization effect

To attribute the change in NDVI to the CO_2_ effect on plant productivity between 1982 and 2015, we used a theoretical relationship that links the increase in photosynthesis to increasing CO_2_^39^ (Eq. 1).

1 $${\mathrm{{GPP}}}_{({\mathrm{{rel}}})} \approx \left[ {\frac{{\left( {c_a - {\Gamma}^ \ast } \right)\left( {c_{a0} + 2{\Gamma}^ \ast } \right)}}{{\left( {c_a + 2{\Gamma}^ \ast } \right)\left( {c_{a0} - {\Gamma}^ \ast } \right)}}} \right]$$

where GPP_(rel)_ is the relative CO_2_ assimilation rate (%), *c*_*a*_ is the atmospheric CO_2_ concentration (µmol mol^−1^), and Γ* is the CO_2_ compensation point in the absence of dark respiration (µmol mol^−1^). We set *c*_*a*0_ to the CO_2_ concentration in 1980^40^ (~339 µmol mol^−1^) and Γ* = 40 (µmol mol^−1^).

Franks et al.^39^ argued that the longer term response of plants to increasing CO_2_ follows the ribulose 1,5-bisphosphate regeneration-limited rate (see also McMurtrie et al.^41^). Accordingly, this relationship implies a conservative response to CO_2_ (as plants may actually follow the Rubisco-limited rate when calculated on a intercellular CO_2_ concentration (*C*_i_) basis during the period of 1982–2015^42^) and ignores any indirect effects (i.e., increased water availability due to stomatal closure, which may extend the growth period in drylands, or interactions with seasonal rainfall^43^). This approach has previously been advocated as a plausible assumption to correctly estimate gross primary productivity (GPP) using satellite light-use efficiency models^44^. We then assume that there has been no change in ratio of GPP to autotrophic respiration (Ra) during this period (1982–2015) and, as a result, the relative change in GPP equates to the relative change in NPP based on Eq. 1. During the period 1982–2015, global air temperatures have risen, which may have led to an increase in Ra^45^. However, increasing temperature has also increased carbon uptake, and both GPP and Ra have been shown to acclimate to the prevailing temperatures^46,47^, meaning that it does not necessarily follow that the GPP : Ra ratio has changed.

We apply the nonlinear CO_2_ relationship (Eq. 1) to the raw NDVI data (NDVI_obs_) to produce a scaled NDVI estimate (NDVI_adj_) that excludes the CO_2_ fertilization effect using Eq. 2 to relate the relative change in NPP to a relative change in NDVI.

2 $$\frac{{\mathrm{{NPP}}}_{{\mathrm{{obs}}}}}{{\mathrm{{NPP}}}_{\mathrm{{base}}}} \approx \frac{{\mathrm{{NDVI}}}_{\mathrm{{obs}}}}{{\mathrm{{NDVI}}}_{\mathrm{{adj}}}}$$

where NPP_obs_ is the NPP at the observed atmospheric CO_2_ concentration (*c*_*a*_), NPP_base_ is the NPP given the same climate conditions but an atmospheric CO_2_ concentration of *c*_*a*0_, NDVI_obs_ is measured NDVI value, and NDVI_adj_ NPP given the same climate conditions but an atmospheric CO_2_ concentration of *c*_*a*0_. Equation 2 was used to calculate a NDVI_adj_ value for every in the full NDVI_obs_ time series with the atmospheric CO_2_ concentrations taken from the IPCC historical forcing data^40^. This approach assumes that NPP and NDVI are linearly related: .. where *b* ≈ 0 and *m* varies spatially. The linear relationship between NPP and NDVI has been observed with both field estimates of NPP^31,32,48^ and estimates of NPP derived from remote-sensing platforms^1,49,50^. However, this assumption of linearity breaks down in densely vegetated regions where NDVI saturates and in biomes with very low above-ground biomass, where the spectral characteristics of the bare soil influences NDVI values^51,52^. As we have excluded hyper-arid and non-water-limited ecosystems, which is a standard practice for studies on desertification (for more information, see ref. ^23^), we expect this assumption of linearity to be robust for our analysis (areas with Aridity index < 0.05 or >0.65 are masked from analysis).

The change in vegetation (ΔNDVI_max_) attributed to the CO_2_ fertilization effect was calculated by first taking the difference between peak growing NDVI with and without CO_2_ fertilization effect (NDVI_obs_ − NDVI_adj_). Similar to the calculation of the observed ΔNDVI_max_, the non-parametric Theil–Sen slope estimator and Spearman’s *ρ* test for significance was then applied to these values. A time-series plot of the mean global NDVI_obs_ and NDVI_adj_ is shown in Supplementary Fig. 7 to highlight temporal nature of this attribution.

### Determining the impact of climate and land use

After the NDVI was scaled to remove the CO_2_ effect using the relationship from Franks et al.^39^, a statistical approach was used to attribute the change in NDVI_adj_ to climate (both CV and CC) and LU. We used the recently developed TSS-RESTREND method, which allows vegetation changes due to LU to be separated from those driven by CC and variability^19,24,53^.

Dryland ecosystems have large natural interannual CV. To separate the impact of climate and LU, previous studies have generally fitted a statistical relationship between climate and vegetation, then used the trends in that relationship or its residuals to quantify LU impacts^54,55^. However, LU can have both a gradual impact on vegetation through processes such as grazing, which are captured by these methodologies^54,56^, and abrupt impact through processes such as deforestation, which cause these methods to break down^18^.

TSS-RESTREND differs from existing dryland trend attribution methods in that it is able to capture both the long-term trends and the step changes in NDVI that occur in regions where ecosystems have experienced significant structural changes^19^. To do this TSS-RESTREND incorporates a phenological change detection method^57^ to identify structural changes in the ecosystem, which manifest as break points in the NDVI time series. We used TSS-RESTREND v2.15, which has been updated to use both precipitation and temperature^24^, to calculate the Vegetation Climate Relationship (VCR) using the per-pixel optimal precipitation and temperature accumulation periods^19,24^. TSS-RESTREND was applied to the NDVI_adj_, with the LU driven component calculated using an ordinary least squared regression between the residuals of the VCR and time, accounting for any detected structural changes to the ecosystem. A similar approach was presented by the IPCC^23^, although that approach does not separate CC from CV and also assumes that all dryland plants follow a C3 photosynthetic pathway.

### Separating climate change and climate variability

The TSS-RESTREND method separates the effects of LU from the combined effects of climate (variability and change) using VCR. To separate the effect of CC and CV, the observed climatology (the per-pixel accumulated precipitation and temperature data) was calculated for the period 1962 to 2015. A 20-year leading edge smoothing window was then applied to this observed climatology to remove the interannual CV. The long-term trend caused by CC was determined using the Theil–Sen slope estimator^58^ applied to the smoothed data and the results were used to detrend the observed climatology.

Using the per-pixel VCR, the total climate driven NDVI (NDVI_CL_) and the NDVI due to CV (NDVI_cv_) were calculated using the observed climatology and detrended climatology, respectively. The difference between NDVI_CL_ and NDVI_CV_ is the change in NDVI_max_ attributable to CC (NDVI_CC_). The non-parametric Theil–Sen slope estimator and Spearman’s *ρ* test for significance was then applied to NDVI_CV_ and NDVI_CC_ to get the change attributable to CV and CC, respectively. The influence of Other Factors (OF), which could not be modeled, was calculated using ${\mathrm{{OF}}} = {\mathrm{{Obs}}} - ({\rm{CO}}_{2} + {\rm{LU}} + {\rm{CV}} + {\rm{CC}})$.

When discussing regional drivers of vegetation change in the main text and in Supplementary Discussion 2, we report the mean climate anomaly rather than the accumulated precipitation and temperature values to allow comparison of different accumulation and offset periods of different pixels. For the observed accumulated precipitation and temperature, the anomaly was calculated on a per-pixel basis using:

3 $$z_n = \frac{{x_n - \mu _{\mathrm{{obs.}}}}}{{\sigma _{\mathrm{{obs.}}}}}$$

where *z* = anomaly, *n* is the year, *x* = observed value, *μ* = mean of the per-pixel accumulated precipitation or temperature, *σ* = SD of the per-pixel accumulated precipitation or temperature. The temperature and precipitation anomaly attributed to CC was calculated using:

4 $$z_n = \frac{{\beta \times (n - n_0)}}{{\sigma _{\mathrm{{obs.}}}}}$$

where *β* is the per-pixel trend in accumulated precipitation or temperature, n_0_ is the first year of the analysis (1982). The temperature and precipitation anomaly attributed to CV was calculated using:

5 $$z_n = \frac{{x_{{\mathrm{{(adj)}}}n} - \mu _{\mathrm{{obs.}}}}}{{\sigma _{\mathrm{{obs.}}}}}$$

where *x*_(adj)_ is the detrended precipitation or temperature. Regional breakdowns of the time series of observed, CC- and CV-driven precipitation and temperature anomaly are shown in Supplementary Figs. 3 and 4, respectively.

### Calculating the impact of Anthropogenic Climate Change

In this study, we use the term ACC to refer to both changes in water availability driven by trends in precipitation and increases in temperature^14,23^, as well as increased water use efficiency (carbon gain per unit of water lost) in response to rising atmospheric CO_2_^8,15^. The impact of ACC) is calculated using ACC = CO_2_ + CC. Although we acknowledge that this considering CO_2_ fertilization and CC together is not common in the literature, these two things are aspects of the same anthropogenic cause; hence, we have reason to discuss them together. It should be noted that although the methodology used in this study is able to capture the effects of other anthropogenic greenhouse gases like methane in the CC component, we cannot separately quantify any direct impact that changes in the amount of these gasses will have on vegetation.

### Accounting for dataset uncertainties and different photosynthetic pathways (C3 vs. C4)

Burrell et al.^53^ showed that using an ensemble of TSS-RESTREND runs using different climate datasets improves the accuracy and minimizes the impact of errors and biases in the individual datasets. Climate data are relatively poorly sampled in dryland regions, which can amplify the documented discrepancies between different datasets^59,60^ We used two 12-member ensembles with matched runs made using TSS-RESTREND analysis performed using every combination of four precipitation and three temperature datasets (see Table 1 for details). The first ensemble assumes all plants are C3 and respond to elevated CO_2_, and the second ensemble assumes all plants are C4 with no eCO_2_ response (see Supplementary Figs. 1 and 2).

**Table 1:** Table of gridded datasets.

Dataset	Description	References
Vegetation		
Global Inventory for Mapping and Modeling Studies (GIMMSv3.1 g)	NDVI, 15 day at 1/16° aggregated to monthly using the max of the valid values	^35^
Precipitation		
University of East Anglia Climate Research Unit TS v. 4.01 (CRU4_p_)	Precipitation, monthly at 0.5°	^72–76^
The Climate Hazards group Infrared Precipitation with Stations v2.0 (CHIRPS)	Precipitation, monthly at 0.05°	^77,78^
Multi-Source Weighted-Ensemble Precipitation (MSWEP)	Precipitation, daily at 0.25°	^79^
TerraClimate	Precipitation, monthly at 1/24°	^80^
Temperature		
University of East Anglia Climate Research Unit TS v. 4.01 (CRU4_T_)	Temperature, monthly at 0.5°	^66–70^
TerraClimate	Temperature, monthly at 1/24°	^80^
National Oceanic and Atmospheric Administration (NOAA) Climate Prediction Center (CPC)	Temperature, daily at 0.5°	^81^
Additional datasets		
TerraClimate	Potential Evapotranspiration (PET) used for the calculation of Aridity index (P/PET)	^80^
Global change in net primary productivity (1981-2003), data from the Food and Agriculture Organization (FAO)	Change in NPP from 1982-2003	^1^
Gridded Population of the World version 4 (GPWv4)	Gridded Population data	^27^
United Nations Development Programme Human Development Index (HDI)	National HDI	^82^
North American Carbon Program (NACP) Global C3 and C4 SYNergetic land cover MAP (SYNMAP)	C3/C4 vegetation fraction	^61^

A third 12-member ensemble, which accounts for the relative fraction of C3 and C4 plants, were calculated by taking the weighted mean of matched runs in ensemble one and two where the weights were the per-pixel fractions of C3 and C4 plants. Estimates of the relative fraction of C4 present in each pixel were derived from the matching 0.5° pixel in the North American Carbon Program (NACP) Global C3 and C4 SYNergetic land cover MAP (SYNMAP)^61^. The *p*-values for each ensemble member were combined using the same weights and the Stouffer’s *Z*-score method. All the results presented in the main paper are from this C4 adjusted ensemble.

We make the assumption that in dryland ecosystems, precipitation controls the amount of foliage cover. As a result, increases in water use efficiency with increasing CO_2_ will lead to increases in foliage cover in plants that use the C3 photosynthetic pathway^8^ while plants that use the C4 photosynthetic pathway are not expected to show this response^62^. Our assumption that C3 plant respond strongly to increased atmospheric CO_2_, and that C4 plants do not respond, which is consistent with theory and short-term CO_2_ enrichment experiments^62^. However, recent surprising results from a long-term CO_2_ manipulation experiment in a dryland ecosystem have shown much higher responses in C4 plants^62^. For this reason, the results of both the C3 and C4 12-member ensembles are included in the Supplementary Material for those interested parties (see Supplementary Figs. 1, 2, 8, and 9).

### Determining statistical significance

For both Obs trend and CO_2_-driven change in NDVI, the Spearman’s rank correlation co-efficient test was used to measure statistical significance for each pixel^63^. In order to determine field significance and account for the multiple testing problem the Benjamini–Hochberg procedure was then applied to these p-values to control the false discovery rate (FDR) (α_FDR_ = 0.10)^64^. As for the uncertainty in the approach we used to measure the CO_2_ fertilization effect, the C3 and C4 12-member ensembles included in the Supplementary Material provide an estimate of the upper and lower bounds of CO_2_ responses (see Supplementary Figs. 1 and 2).

For LU, CV, and CC there are 12 members in each ensemble. The *p*-values of these members were combined using the Fisher’s combined probability test and then the Benjamini–Hochberg procedure was applied to these *p*-values to determine statistical significance (*α*_FDR_ = 0.10). In addition, we also applied the IPCC protocol for determining ensemble significance and agreement^65^. For a pixel to be significant under this protocol, >50% of ensemble members must find a significant change (*α*_FDR_ = 0.10) and, of those significant runs, >80% must agree on the direction of change. If a pixel fails either the overall significance test or the IPCC protocol, the estimate of that component is masked for that pixel. Similar criteria have also been applied to ensemble breakpoint detection (>50% of runs must find a significant breakpoint, 80% of which must be in a three-year window) the results of which are included in supplementary material.

All the Climate datasets where interpolated from their native resolution to the 1/12° grid of the GIMMSv3.1 g datasets using the First-Order Conservative Remapping^66^ in CDO^67^. Additional datasets where used to aid the interpretation and discussion our results. All dataset where converted to the GIMMS grid to allow for per-pixel comparison. To separate CC from CV, a 20-year leading edge moving window was used where the value for a given year is the mean of the previous 20 years. For this reason, it was necessary to have climate data that goes back to 1960, which not all datasets do. Those climate datasets with insufficient temporal record were extended using TERRACLIMATE precipitation and temperature, and the Delta Bias Correction method^68^.

## Supplementary information

Supplementary Information

Peer Review File

## Data Availability

GIMMS NDVI data can be accessed using the gimms R-package [https://cran.r-project.org/web/packages/gimms/] or on request to the dataset authors^35^. CRU climate data are available from [http://data.ceda.ac.uk//badc/cru/data/cru_ts/] with the identifier [Dataset DOI: http://doi.org/10/gcmcz5]. CHIRPS data can be accessed from [ftp://chg-ftpout.geog.ucsb.edu/pub/org/chg/products/CHIRPS-2.0/] with the identifier [10.1038/sdata.2015.66 2015]. The MSWEP climate data are available from [http://www.gloh2o.org/] or on request to the dataset authors^69^. TERRACLIMATE data are available from [http://www.climatologylab.org/terraclimate.html] with the identifier [10.1038/sdata.2017.191]. CPC temperature data can be accessed from [ftp://ftp.cdc.noaa.gov/Datasets/cpc_global_temp/].

## Code availability

The full per-pixel attribution method used in this paper is available in v0.3.0 of the TSS-RESTREND R-package, which is available from [https://cran.r-project.org/web/packages/TSS.RESTREND/index.html]. Scripts showing how to apply this method to spatial data, calculate ensemble statistics, and produce maps can be accessed at [https://github.com/ArdenB/TSSRESTREND]. Statistical tests were performed using the statsmodels python package [https://www.statsmodels.org/] and the scikit-learn python package^70^. All maps were produced using the Cartopy Python package^71^, which uses the public domain datasets available from [http://www.naturalearthdata.com] for coastlines, rivers, and national borders. All additional code related to batch processing is specific to the High Performance Computing Environment used to perform the analysis but is available on reasonable request to the authors.
