# Supplementary material for: Anthropogenic climate change has driven over 5 million km2 of drylands towards desertification
Source: Nat Commun. 2020 Jul 31;11:3853. doi: 10.1038/s41467-020-17710-7 (PMC7395722; doi:10.1038/s41467-020-17710-7)
Supplement: Supplementary file 1 — Supplementary Information [file 41467_2020_17710_MOESM1_ESM.pdf]

Supplementary Information for Anthropogenic Climate change has driven over 5 million km^2 of drylands towards desertification by Burrell et al.

Supplementary Text

1. *Comparing our findings with global studies*

In the main part of this article we compare our results to two recent global studies by Song et al.,[1] and Zhu et al.,[2] which have examined the drivers of global vegetation change. Zhu et al.,[2] used a 10 model ensemble with change attribution performed by running models with and without different drivers and then comparing the results. Song et al.,[1] used AVHRR derived vegetation fractions to look at change in vegetation types, then performed attribution using high resolution imagery at 1500 locations across the globe. At each location, Song at al.,[1] looked for "*visible signs of human activity*" and if these signs where present then the observed change was attributed to land use and if not then it was attributed to "*indirect drivers such as climate change*".

These two studies highlight the existing inconsistency in the published literature, with the literature showing that the broad trends in vegetation change are consistent but there are large differences in attribution. The comparison between our study and these studies is not perfect due to different spatial domains. Our study only looks at the dryland biomes, with these making up only ~50% of land area that these global studies cover. Both global studies found that the importance of drivers varies between biomes. This section provides additional discussion and support for our argument that that the underlying assumptions made, and methods used in the attribution of change, are the source of much of the discrepancy between our findings and these previous studies.

1.1 T*he CO_2 fertilisation effect*

The impact of CO_2 in drylands is well documented[3] and as discussed in the main text, Song et al.,[1] does not attribute change to CO_2 which leads to Song et al., identifying Land Use and Climate as the dominate drivers of change. Supplementary Figure 1 shows a detailed breakdown of the results of our attribution approach if we assumed no CO_2 fertilisation effect. An example of this can be seen in eastern China where Song et al.,[1] finds land use driven change. Our C3 ensemble, which ignores CO_2 comes to the same conclusion. However, our mixed C3/C4 ensemble which includes CO_2 finds that the CO_2 is the dominant driver in this region (Figure 1b and Supplementary Figure 8c). Similarly, in the southern part of South America, our C3 ensemble which ignores CO_2 finds Climate Change and Climate Variability to be the dominant drivers, (Supplementary Figure 1 and Supplementary Figure 8c) which is consistent with Song et al.,[1]. However, when CO_2 is included, large areas of negative land use become apparent (Figure 4d and Supplementary Figure 8a). Our conclusion that CO_2 fertilisation is the dominate driver of vegetation change, is consistent with the findings of Zhu et al.,[2]. Figure 1b does not show areas where 'Other Factors' was dominant to be comparable to Song et al.,[1]. For interested parties, Supplementary Figure 8 shows the dominant driver in all three ensembles with Other Factors included which is more comparable to the result of Zhu et al.,[2].

1.2 *Land use*

When comparing the attribution performed by Song et al.,[1] to the Land Use changes detected by our approach (Figure 4d), there was generally broad agreement between large clusters of points. For example, in India and China where Song et al., found change was dominated by Land Use , we also found large land use driven changes, though in some places the changes attributed to Land Use were smaller in magnitude than the changes driven by CO_2. This highlights the value of quantifying the impact and direction of all the drivers, not just the dominant one. Directly comparing our findings is not possible in all regions as the point-based attribution approach used by Song et al.,[1] has large gaps between attribution sites in places like Mongolia. In Mongolia there is strong evidence that grazing is the main Land Use impact [4], a result supported by our attribution approach which finds Land Use to be the dominant driver.

These findings differ considerably from the results of Zhu et al.,[2] who found a significantly smaller Land Use effect. The models used by the Zhu et al., study do not calculate the land use changes but instead rely on prescribed datasets to incorporate processes like deforestation [2,5–9]. Existing work has shown that models are very sensitive to the land use data used to force them [10,11]. The data used to drive the TRENDY models used by Zhu et al., (the HYDE dataset) is national data downscaled to the model's grid and does not include many important processes like grazing which is dominant land use in many dryland region's [2,6,7]. An example of this issue can be seen when looking at Mongolia where grazing of goats is known to be driving widespread change [4].

In this region we find large areas where Land Use is the dominate driver of change, while Zhu et al., attributes change in the region to Other Factors. Furthermore, the way the TRENDY models implement Land Use varies significantly which leads the estimates to *"differ significantly in magnitude, and sometimes also in sign"*[2]. The tendency for modelling approaches to underestimate Land Use has been documented by several recent studies[22,23].

1.3 Climate change and variability

By definition, dryland areas are very sensitive to water availability and are predominantly found in areas with high natural climate variability[12]. Climate Change and Climate Variability is another area where our findings differ significantly from the results of Zhu et al.,[2]. The TRENDY models are not able to measure climate variability and the estimate of climate change impacts vary considerably between models[2]. A good example of the difference between our findings and that of Zhu et al.,[2] can be seen in the Sahel where Zhu et al., attribute much of the change in vegetation to Climate Change, while our findings, as well as previous work in that region, attribute it to decadal climate variability (see Supplementary Text 1.1). The approach used by Song et al.,[1] does not distinguish between climate change and climate variability. Previous studies have raised serious concerns about the validity of trend detection and attribution in dryland vegetation, especially in short vegetation like grasses, that is performed without properly accounting for natural climate variability[12–14].

1.4 Nitrogen deposition

Nitrogen deposition is driver of vegetation change that our approach was not able to quantify but was included in the Zhu et al.,[2]analysis. Zhu et al.,[2] found nitrogen deposition to be the second largest driver globally, but noted that this finding was uncertain given that only two of their models could be run with and without the nitrogen deposition. There is good evidence that nitrogen deposition is not an important driver of vegetation change in drylands. A recent meta-analysis on the impacts of nitrogen deposition on plant species found that drylands are not sensitive to increased N loads because they are overwhelmingly water limited and, with the exception of a small part of the southwestern United States, *"are mainly present in regions with very low N deposition"* [15] This conclusion is supported by the findings of Zhu et al.,[2] who found that Nitrogen played little to no role in dryland areas.

2.5 Fires

The impact of fire on long-term dryland vegetation change is not quantified by our method or by either of the global studies undertaken by Song et al., and Zhu et al. Dryland fires are an important contributing factor to the carbon cycle and on atmospheric aerosols [16]. The natural fire return interval in drylands is very short, with many dryland tree species having evolved adaptations, such as epicormic resprouting (the rapid reestablishment of extensive leaf area) to survive and recover after fires[17]. The grasses and shrubs are also adapted to recover rapidly after fires. This rapid recovery is not apparent in all ecosystems, with growing evidence that the dry forests in the alpine parts of the southwestern United States are experiencing fire-driven desertification [18]. Similarly, when developing the TSS-RESTREND method used in this study, Burrell et al., [14] found that while fire did not seem to impact long term vegetation trends or change the ecosystem structure in most locations, in the southeastern alpine zone extreme fire events had caused permanent forest loss. In this case, TSS-RESTREND detects the change and attributes it to Land Use.

2. *Validating our findings against regional studies*

In this section we discuss our findings compared to regional studies. When discussing the impact of climate change we report the regional means of the accumulated precipitation anomaly (APA) and accumulated temperature anomaly (ATA) driven by climate change and climate variability. The calculation of these anomalies is described in the methods and a full regional time-series is included in Supplementary Figure 3 and Supplementary Figure 4.

2.1. Hotspots of desertification

a. Central and Western Asia

Over parts of central and western Asia, primarily Turkmenistan, Kazakhstan and Uzbekistan though to the southern border of Russia, we observe widespread desertification driven mostly by land use and to a lesser extent climate change (see Figure 4 and **Supplementary Figure 2**). In this region there is documented evidence of long-term degradation driven by unsustainable land use practices, widespread farm abandonment after the breakup of the Soviet Union and the over utilisation of water resources resulting in soil salinization [19–21]. These negative drivers have been compounded by a long-term climate change driven increase in temperature (mean ATA_{CC} ≈ 1 from 1982 to 2015) resulting in the so called 'Aral Sea disaster' [22]. We found large clusters of breakpoints in this regions (**Supplementary Figure 5**) which is consistent with previous findings [23] though methodological and data variations make a direct validation of timings difficult. Similarly, over the Middle East we observed negative impacts of climate change and land use (Figure 4) which is consistent with regional studies that have documented the impact of climate change intensification of droughts [24] which has been amplified by unsustainable agriculture and ground water extraction [25–28]. The Middle East and the western part of Central Asia was responsible for almost ¾ of the global net permanent water loss [29].

b. South America

The largest hotspot of desertification in South America is the semi-arid Caatinga forest in north-western Brazil. In this region, we observed negative changes in both the climate change and land use components (Figure 4). In this region, widespread deforestation and grazing intensification has compounded the effect of a long term decline in precipitation [30–34] driven by climate change (mean APA_{CC} ≈ -0.2 from 1982 to 2015) which has led to widespread desertification [35]. The second hotspot of desertification in South America is over Argentina. Our results suggest that this change is predominantly driven by unsustainable land use which is consistent with some regional studies [36,37], though a more thorough validation is currently difficult because of documented issues in the publishing of desertification studies from this region [38].

2.2. Greening hotspots

a. Sahel

The greening of the Sahel has also been the focus of a large number of both remote sensing and field observation studies [39]. Over the Sahel we find the vegetation greening ($\Delta NDVI_{max}$: 0.050 ± 0.052) is dominated by CO_2 ($\Delta NDVI_{max}$: 0.018±0.007), climate variability ($\Delta NDVI_{max}$: 0.012±0.017) and land use ($\Delta NDVI_{max}$: 0.010±0.028) which play larger roles than climate change ($\Delta NDVI_{max}$:0.005±0.0249) (Figure 3). Our attribution of greening over the Sahel, is consistent with the conclusion that the greening is driven by increased rainfall caused by the wet phase of the natural decadal variability reinforced by the CO_2 effect [39–45]. The shift from the dry phase to the wet phase of climate variability can be seen in Supplementary Figure 3. Between 1982 and 1988 the Sahel had below average rainfall (mean APA_{cv} ≤ 0) including 1984 which had the largest negative precipitation anomaly observed in any region over the period 1982 to 2015 ($APA_{cv(1984)}$ ≈ -1.1). In contrast, the only year from the period 2010 to 2015 with below average precipitation was 2011 with and APA_{CV} ≈ -0.1.

b. India

The greening we observed over India was the largest of any region and has been observed by other studies [46]. Since 2000, there has been a well-documented revival of the Indian summer monsoon which has resulted in increased rainfall[47] that can be seen in Supplementary Figure 3. This, combined with changes in groundwater policy and better irrigation practices has led to agricultural intensification [48–51]. This is consistent with our findings that land use ($\Delta NDVI_{max}$: 0.036 ± 0.043), CO_2 ($\Delta NDVI_{max}$: 0.028 ± 0.011) and climate variability ($\Delta NDVI_{max}$: 0.016 ± 0.017) are the dominate drivers of change in the region (Figure 3). India is the only region where land use had a larger mean positive per pixel contribution than CO_2.

c. Australia

Australia experienced greening despite the largest negative climate change impact of any region (-0.010 ± 0.028) with the greening in this region driven by CO_2 (0.018 ± 0.010), land use (0.015 ± 0.032) and climate variability (0.010 ± 0.023) (Figure 3). The dominance of CO_2 is consistent with previous findings [3]. There is evidence that the positive land use component is being driven by both agricultural intensification as well as ecosystem recovery after the release of a viral biological control that killed 95% of Australia's feral rabbits [14,52].

While a lack of large scale field observations makes this hard to confirm, there is evidence for the recovery of both vegetation and native animal populations [53–56]. The evidence for the impact of climate variability is well documented in Australia. From 2001 to 2009 much of Australia experienced the Millennium Drought[57] which was characterised by below average rainfall and above average temperatures (Supplementary Figure 3 and Supplementary Figure 4). The drought came to an end in 2010-2011 with extreme precipitation associated with a strong La Niña event which is the largest positive precipitation anomaly observed in any region over the period 1982 to 2015 ($APA_{cv} \approx 1.1$). This rainfall caused a massive increase in vegetation productivity with Australia accounting for 60% of the global land carbon sink that year[58].

d. Eastern Asia

The greening in eastern Asia is mostly concentrated in northern and central China which we attribute mostly to land use (Figure 4). The increase in vegetation over China is well documented and is the result of the implementation of reforestation projects and occurred despite negative effects of climate change and variability [59,60]. This provides a case study of how land use intervention can be used to offset the negative impacts of climate change and variability.

1.3 The influence of drivers in regions at risk of desertification

Given the positive forcing of the CO_2 increase it is perhaps surprising that >50 % of dryland areas have not significantly changed. In these regions, negative changes in the land use and climate change components are being counterbalanced by the CO_2 fertilisation effect and the positive phase of natural climate variability. These areas are at high risk of desertification because climate stress and land use pressure can compound and it would only take a small increase in the negative effect of these drivers or a shift to the negative phase of natural climate variability to cause a significant negative change [61]. One example of this is over the dryland regions of Southern Africa where unsustainable land use and negative climate change effects are masked by CO_2 and climate variability (Figure 4). This has important consequences for many countries in the region, as they are among the least food secure in the world. As an example, in Zambia, a country where the CO_2 adjusted analysis found wide spread negative climate change and land use components entirely missed by the non-adjusted analysis, the United Nations World Food Programme estimates that 40% of children under 5 are stunted due to malnutrition [62].

Supplementary Figures

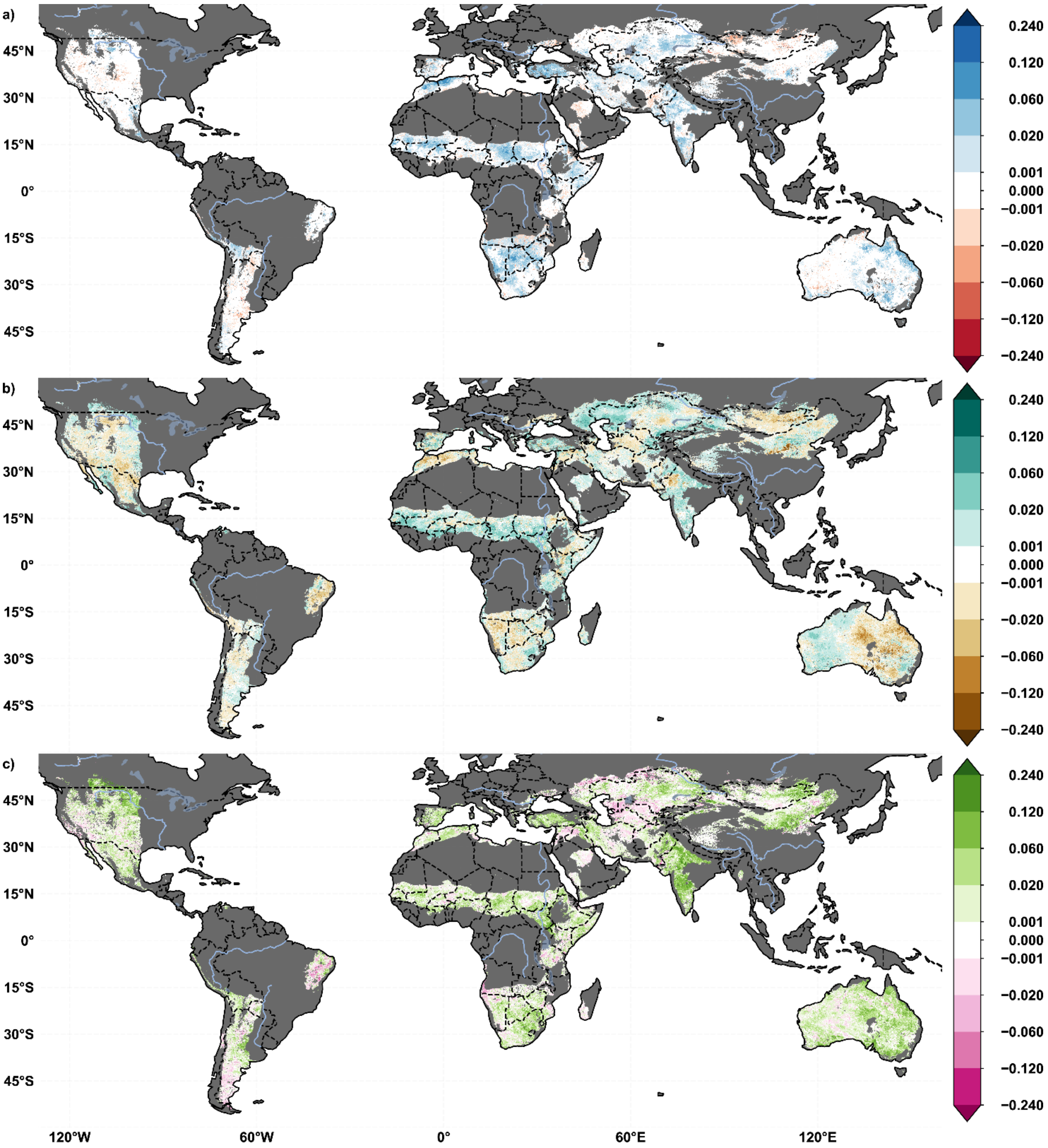

Supplementary Figure 1 The Drivers of Global Vegetation Change assuming a C4 photosynthetic mechanism. The changes in NDVImax between 1982 and 2015 ($\Delta NDVI_{max}$) attributed to a) climate variability b) climate change and c) land use assuming all plants follow a C4 pathway with no response to eCO_2. Non-dryland regions are masked in dark grey. Areas where the change did not meet the multi-run ensemble significance criteria detailed in the methods or are smaller than the error in the sensors (±0.001) are masked in white.

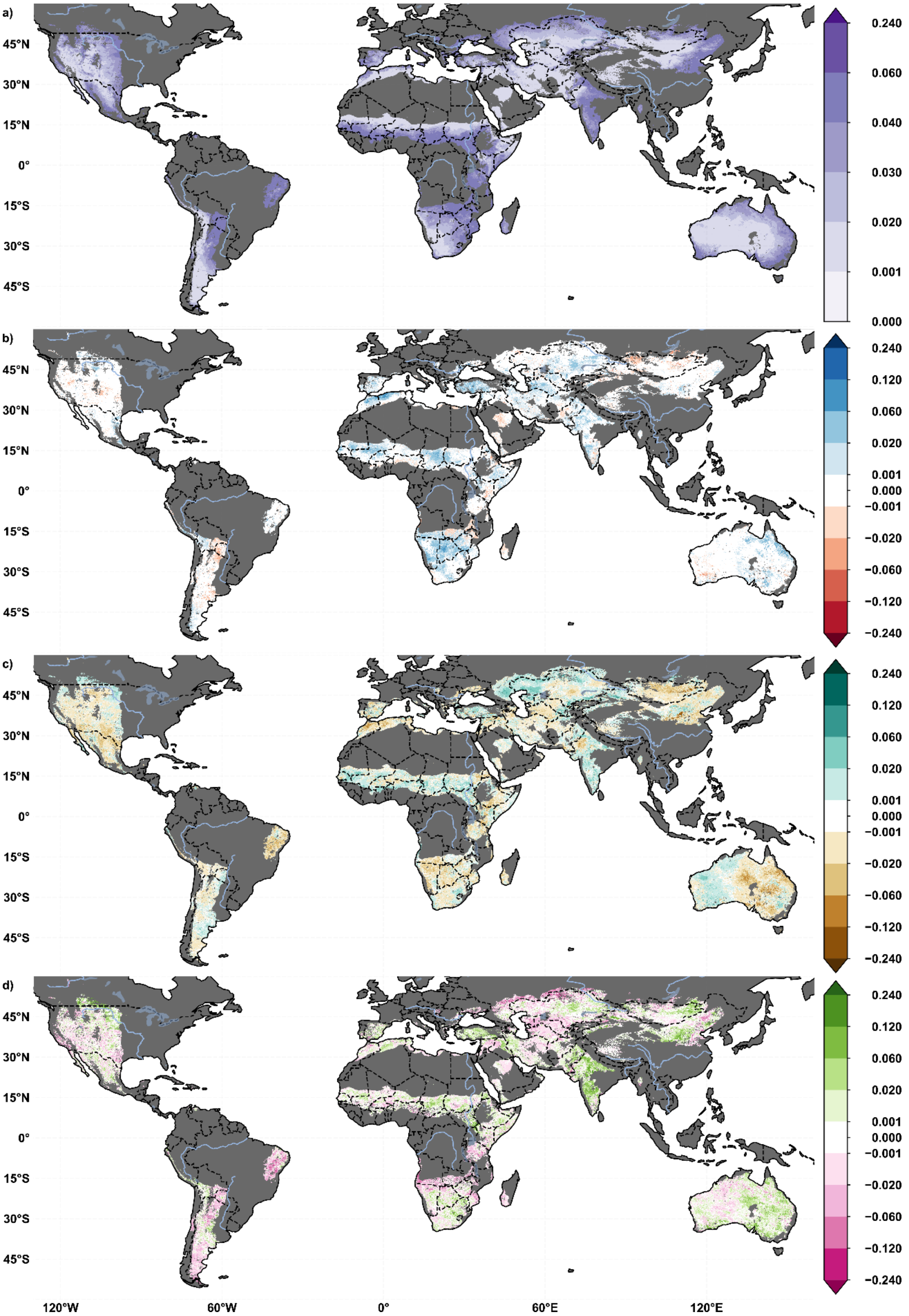

Supplementary Figure 2 The Drivers of Global Vegetation Change assuming a C3 photosynthetic mechanism. The changes in $NDVI_{max}$ between 1982 and 2015 ($\Delta NDVI_{max}$) attributed to a) CO_2 fertilisation b) climate variability c) climate change and d) land use assuming all plants follow a C3 response to eCO_2. Non-dryland regions are masked in dark grey. Areas where the change did not meet the multi-run ensemble significance criteria detailed in the methods or are smaller than the error in the sensors (±0.001) are masked in white.

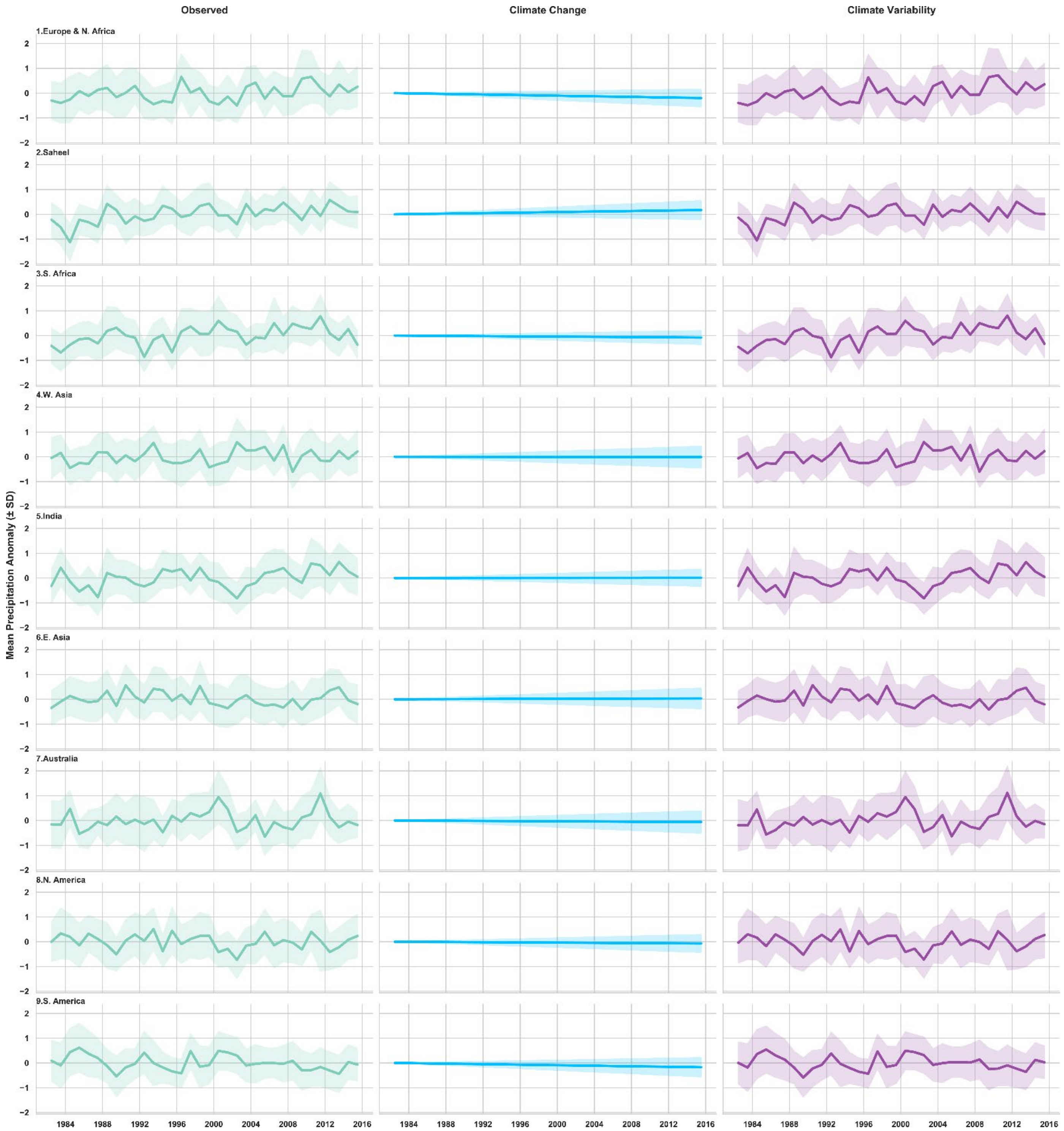

Supplementary Figure 3 Regional precipitation anomaly and its driver's. The solid lines shows the observed (teal), climate change driven (blue) and climate variability driven (purple) mean per pixel (± 1 standard deviation) temperature anomaly from 1982 to 2015 broken out by region.

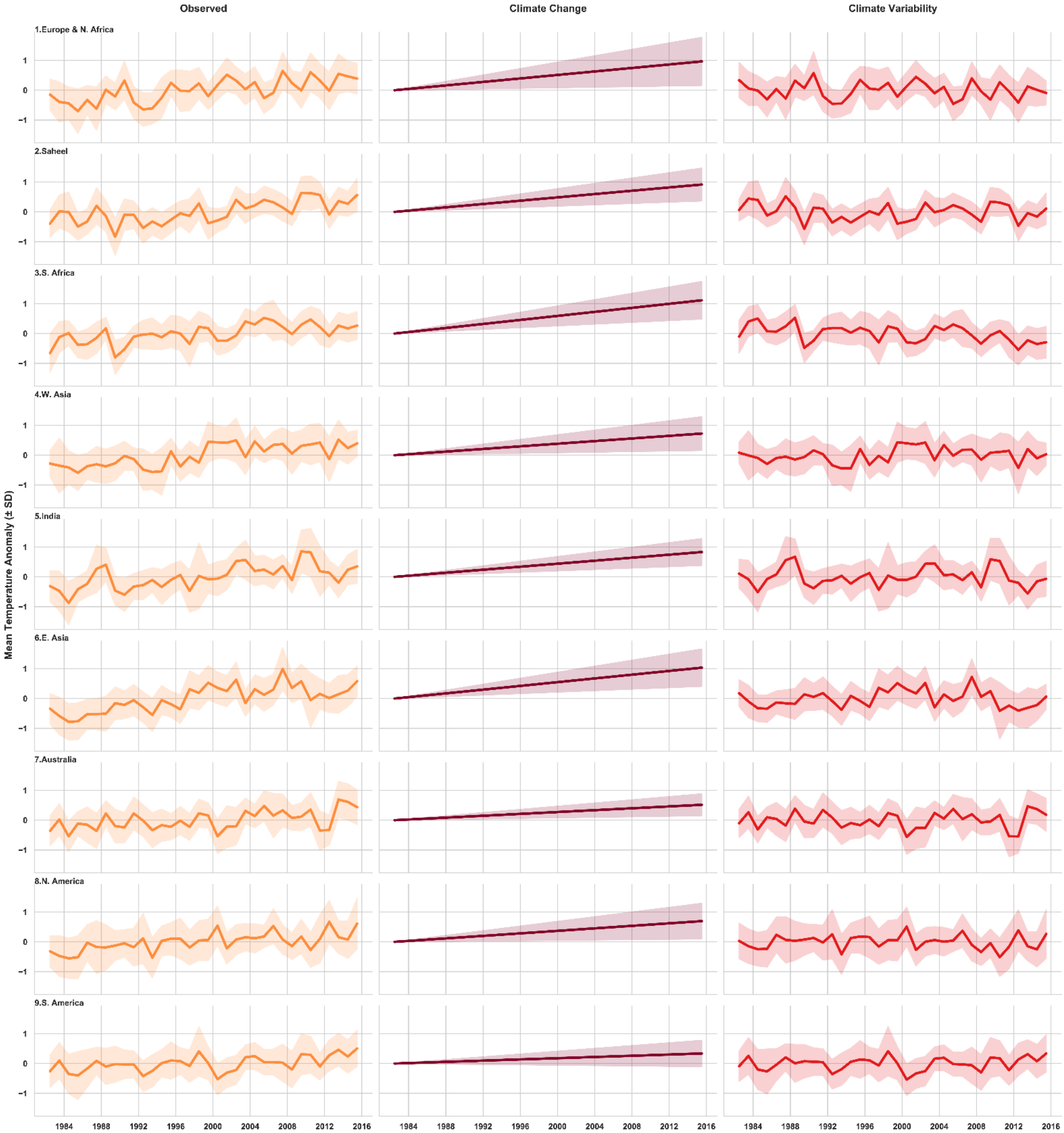

Supplementary Figure 4 Regional temperature anomaly and its driver's. The solid lines shows the observed (orange), climate change driven (maroon) and climate variability driven (red) mean per pixel (± 1 standard deviation) temperature anomaly from 1982 to 2015 broken out by region.

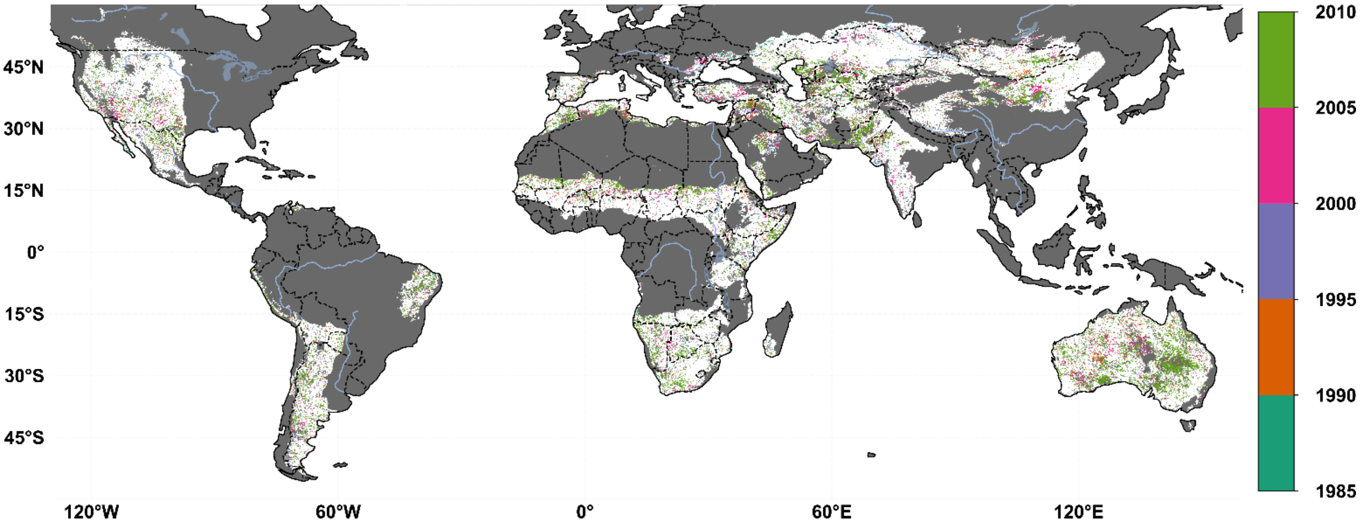

Supplementary Figure 5 The timing of breakpoints in the mixed C3/C4 ensemble. To determine if a breakpoint was significant in the ensemble, > 50% of runs had to have a breakpoint of which >80% had to fall within a three-year window.

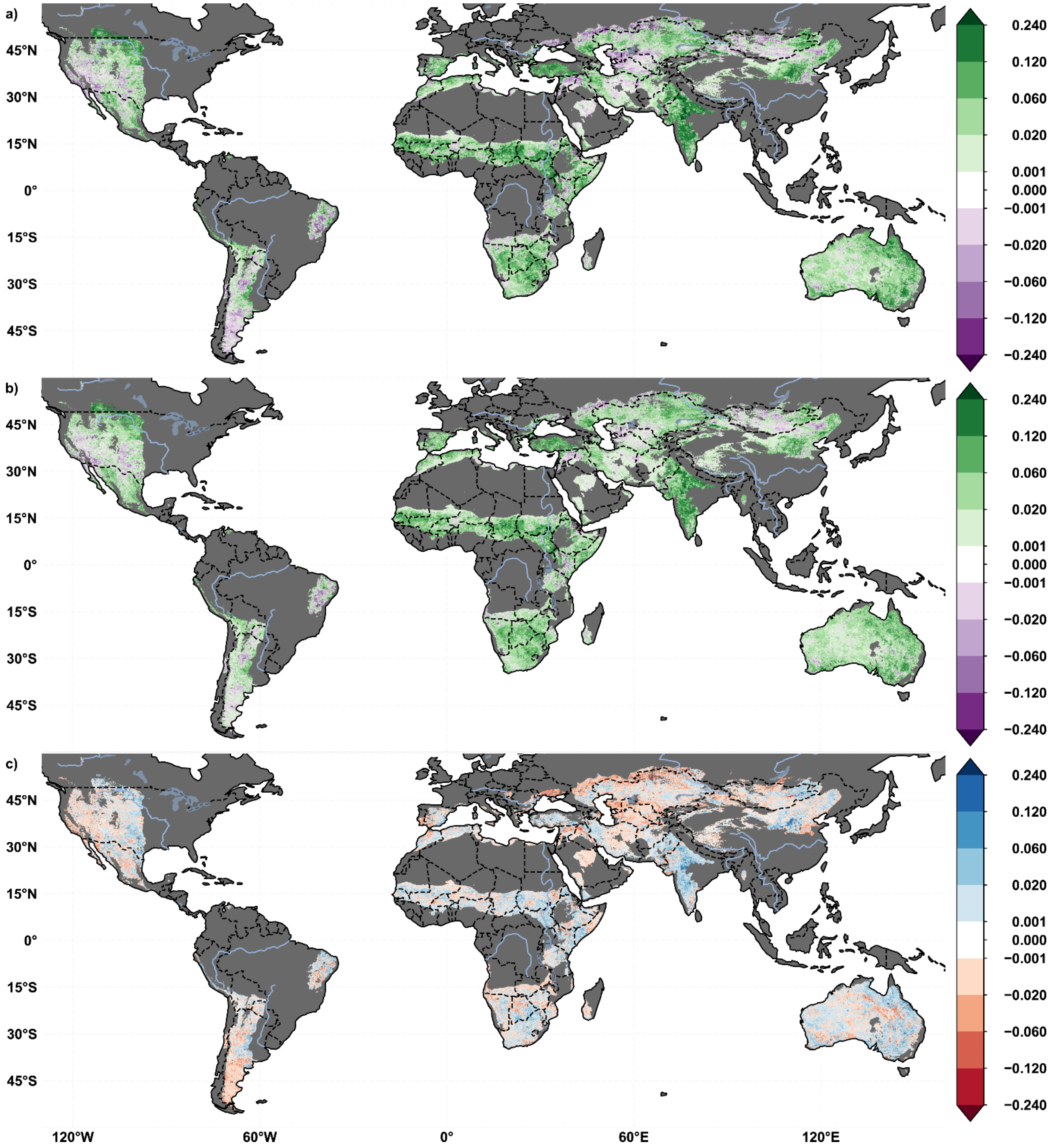

Supplementary Figure 6 Comparison of observed vegetation change to the attributable change assuming a mixed C3/C4 response. a) the observed change in vegetation ($\Delta NDVI_{max}$) between 1982 and 2015 (Obs) b) The sum of the attributable change (CO_2 + CC + CV + LU). c) the difference between the observed vegetation change and the attributable vegetation change (OF = Obs - (CO_2 + CC + CV + LU)). $\Delta NDVI_{max}$ smaller than the error in the sensors (±0.001) are masked in white.

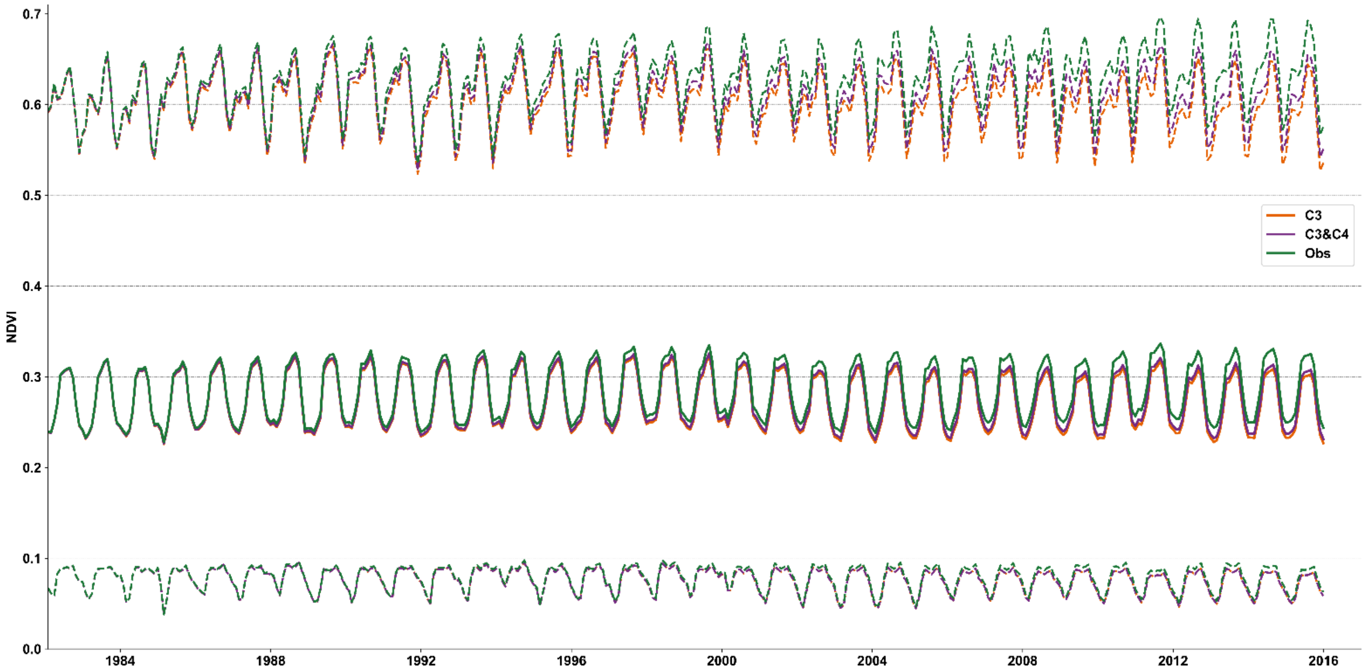

Supplementary Figure 7 Monthly mean global dryland GIMMS NDVI values. The Solid lines are the global monthly mean NDVI values while the upper and lower dotted lines represent the 95th and 5th percentile respectively. The green line is the observed values ($NDVI_{obs}$) while the purple and orange lines represent the NDVI values after they have been adjusted to remove the CO_2 fertilisation effect ($NDVI_{adj}$).

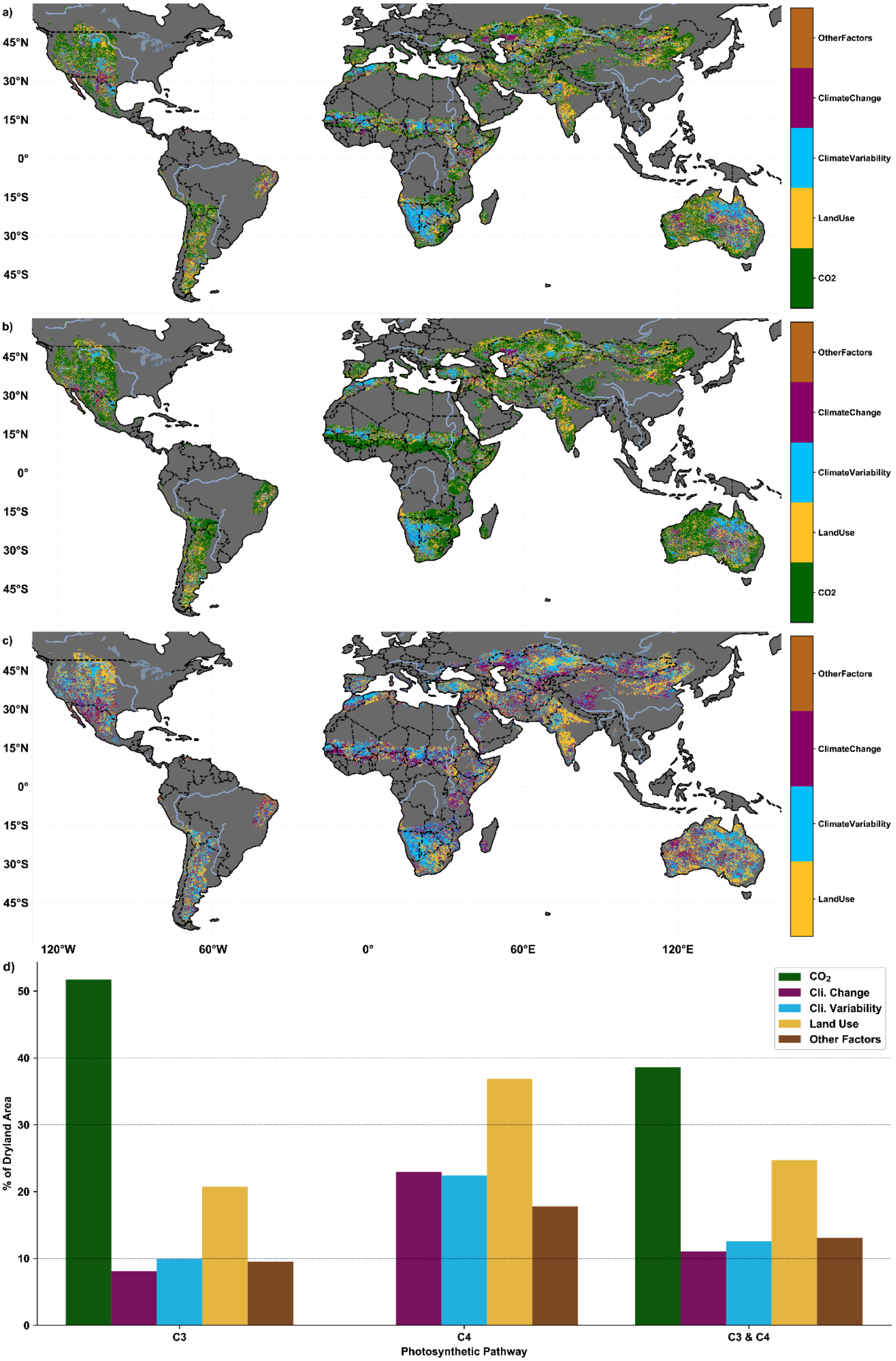

Supplementary Figure 8 Primary drivers of vegetation changes between 1982 and 2015 a-c) Maps showing the largest absolute driver (CO_2, land use, climate change, climate variability and other factors) assuming a) assuming a mixed C3/C4 photosynthetic mechanism b) assuming a C3 photosynthetic mechanism c) assuming a C4 photosynthetic mechanism. Non-dryland regions are masked in dark grey d) Largest absolute driver by percentage of global dryland area assuming a C3, C4 and mixed C3/C4 photosynthetic mechanism,

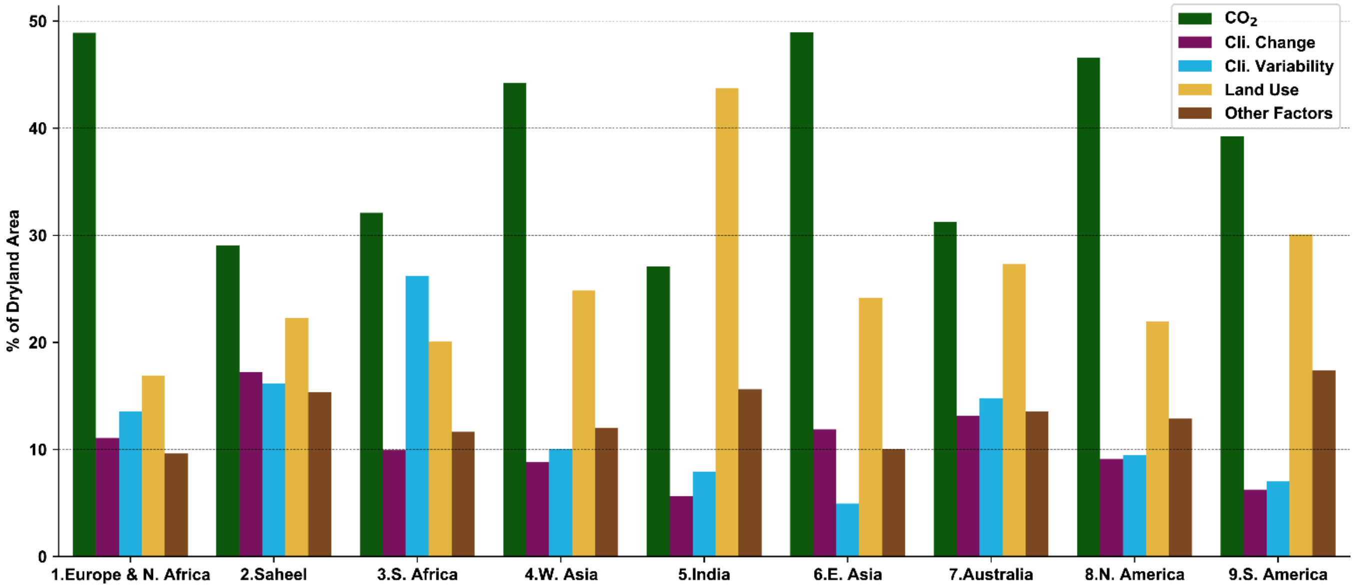

Supplementary Figure 9 Primary regional drivers of vegetation change. Largest absolute driver by percentage of regional dryland area assuming a mixed C3/C4 photosynthetic mechanism. This figure is a regional breakdown of the information show in Supplementary Figure 3a.

Supplementary References

1. Song, X.-P. *et al.* Global land change from 1982 to 2016. *Nature* 1 (2018) doi:10.1038/s41586-018-0411-9.
2. Zhu, Z. *et al.* Greening of the Earth and its drivers. *Nat. Clim. Change* **6**, 791 (2016).
3. Ukkola, A. M. *et al.* Reduced streamflow in water-stressed climates consistent with CO2 effects on vegetation. *Nat. Clim. Change* **6**, 75–78 (2016).
4. Liu, Y. Y. *et al.* Changing Climate and Overgrazing Are Decimating Mongolian Steppes. *PLoS ONE* **8**, e57599 (2013).
5. Calle, L. *et al.* Regional carbon fluxes from land use and land cover change in Asia, 1980–2009. *Environ. Res. Lett.* **11**, 074011 (2016).
6. Li, W. *et al.* Land-use and land-cover change carbon emissions between 1901 and 2012 constrained by biomass observations. *Biogeosciences* **14**, 5053–5067 (2017).
7. Peng, S. *et al.* Sensitivity of land use change emission estimates to historical land use and land cover mapping: Land Use and Land Cover Mapping. *Glob. Biogeochem. Cycles* **31**, 626–643 (2017).
8. Tharammal, T., Bala, G., Devaraju, N. & Nemani, R. A review of the major drivers of the terrestrial carbon uptake: model-based assessments, consensus, and uncertainties. *Environ. Res. Lett.* **14**, 093005 (2019).
9. Yue, C. *et al.* Representing anthropogenic gross land use change, wood harvest, and forest age dynamics in a global vegetation model ORCHIDEE-MICT v8.4.2. *Geosci. Model Dev.* **11**, 409–428 (2018).
10. Arneth, A. *et al.* Historical carbon dioxide emissions caused by land-use changes are possibly larger than assumed. *Nat. Geosci.* **10**, 79–84 (2017).
11. Bayer, A. D. *et al.* Uncertainties in the land-use flux resulting from land-use change reconstructions and gross land transitions. *Earth Syst. Dyn.* **8**, 91–111 (2017).
12. Intergovernmental Panel on Climate Change (IPCC). *Chapter 3: Desertification in the IPCC Special Report on Climate Change, Desertification, Land Degradation, Sustainable Land Management, Food Security, and Greenhouse gas fluxes in Terrestrial Ecosystems.* https://www.ipcc.ch/site/assets/uploads/2019/08/2d.-Chapter-3_FINAL.pdf (2019).
13. Wessels, K. J., van den Bergh, F. & Scholes, R. J. Limits to detectability of land degradation by trend analysis of vegetation index data. *Remote Sens. Environ.* **125**, 10–22 (2012).
14. Burrell, A. L., Evans, J. P. & Liu, Y. Detecting dryland degradation using Time Series Segmentation and Residual Trend analysis (TSS-RESTREND). *Remote Sens. Environ.* **197**, 43–57 (2017).
15. Bobbink, R. *et al.* Global assessment of nitrogen deposition effects on terrestrial plant diversity: a synthesis. *Ecol. Appl.* **20**, 30–59 (2010).
16. Rogers, B. M., Balch, J. K., Goetz, S. J., Lehmann, C. E. R. & Turetsky, M. Focus on changing fire regimes: interactions with climate, ecosystems, and society. *Environ. Res. Lett.* **15**, 030201 (2020).
17. Burrows, G. E. Buds, bushfires and resprouting in the eucalypts. *Aust. J. Bot.* **61**, 331–349 (2013).
18. Neary, D. G. Wildfire contribution to desertification at local, regional, and global scales [Chapter 8]. *Squires Victor Roy Ariapour Ali Eds Desertification Desertification Past Curr. Future Trends Hauppauge NY Nova Sci. Publ. Inc P 199-222* 199–222 (2018).
19. Alcantara, C. *et al.* Mapping the extent of abandoned farmland in Central and Eastern Europe using MODIS time series satellite data. *Environ. Res. Lett.* **8**, 035035 (2013).
20. Lesiv, M. *et al.* Spatial distribution of arable and abandoned land across former Soviet Union countries. *Sci. Data* **5**, 180056 (2018).
21. Robinson, S. Land Degradation in Central Asia: Evidence, Perception and Policy. in *The End of Desertification? : Disputing Environmental Change in the Drylands* (eds. Behnke, R. & Mortimore, M.) 451–490 (Springer Berlin Heidelberg, 2016). doi:10.1007/978-3-642-16014-1_17.
22. Lioubimtseva, E. A multi-scale assessment of human vulnerability to climate change in the Aral Sea basin. *Environ. Earth Sci.* **73**, 719–729 (2015).
23. Horion, S. *et al.* Revealing turning points in ecosystem functioning over the Northern Eurasian agricultural frontier. *Glob. Change Biol.* **22**, 2801–2817 (2016).

24. Cook, B. I., Anchukaitis, K. J., Touchan, R., Meko, D. M. & Cook, E. R. Spatiotemporal drought variability in the Mediterranean over the last 900 years. *J. Geophys. Res. Atmospheres* **121**, 2060–2074 (2016).
25. Abahussain, A. A., Abdu, A. Sh., Al-Zubari, W. K., El-Deen, N. A. & Abdul-Raheem, M. Desertification in the Arab Region: analysis of current status and trends. *J. Arid Environ.* **51**, 521–545 (2002).
26. Joodaki, G., Wahr, J. & Swenson, S. Estimating the human contribution to groundwater depletion in the Middle East, from GRACE data, land surface models, and well observations. *Water Resour. Res.* **50**, 2679–2692 (2014).
27. Schaldach, R. *et al.* Model-based analysis of the environmental impacts of grazing management on Eastern Mediterranean ecosystems in Jordan. *J. Environ. Manage.* **127**, S84–S95 (2013).
28. Zafarnejad, F. The contribution of dams to Iran's desertification. *Int. J. Environ. Stud.* **66**, 327–341 (2009).
29. Pekel, J.-F., Cottam, A., Gorelick, N. & Belward, A. S. High-resolution mapping of global surface water and its long-term changes. *Nature* **540**, 418–422 (2016).
30. Marinho, F. P., Mazzochini, G. G., Manhães, A. P., Weisser, W. W. & Ganade, G. Effects of past and present land use on vegetation cover and regeneration in a tropical dryland forest. *J. Arid Environ.* **132**, 26–33 (2016).
31. Novaes, R. L. M., Felix, S. & Souza, R. de F. Brazil: Save Caatinga from drought disaster. *Nature* https://www.nature.com/articles/498170a (2013) doi:10.1038/498170a.
32. Ribeiro, E. M. S., Arroyo-Rodríguez, V., Santos, B. A., Tabarelli, M. & Leal, I. R. Chronic anthropogenic disturbance drives the biological impoverishment of the Brazilian Caatinga vegetation. *J. Appl. Ecol.* **52**, 611–620 (2015).
33. Mariano, D. A. *et al.* Use of remote sensing indicators to assess effects of drought and human-induced land degradation on ecosystem health in Northeastern Brazil. *Remote Sens. Environ.* **213**, 129–143 (2018).
34. Lausier, A. M. & Jain, S. Overlooked Trends in Observed Global Annual Precipitation Reveal Underestimated Risks. *Sci. Rep.* **8**, 1–7 (2018).
35. Tomasella, J. *et al.* Desertification trends in the Northeast of Brazil over the period 2000–2016. *Int. J. Appl. Earth Obs. Geoinformation* **73**, 197–206 (2018).
36. Busso, C. A. & Fernández, O. A. Arid and Semiarid Rangelands of Argentina. in *Climate Variability Impacts on Land Use and Livelihoods in Drylands* (eds. Gaur, M. K. & Squires, V. R.) 261–291 (Springer International Publishing, 2018). doi:10.1007/978-3-319-56681-8_13.
37. Chartier, M. P., Rostagno, C. M. & Pazos, G. E. Effects of soil degradation on infiltration rates in grazed semiarid rangelands of northeastern Patagonia, Argentina. *J. Arid Environ.* **75**, 656–661 (2011).
38. Torres, L., Abraham, E. M., Rubio, C., Barbero-Sierra, C. & Ruiz-Pérez, M. Desertification Research in Argentina. *Land Degrad. Dev.* **26**, 433–440 (2015).
39. Kaptué, A. T., Prihodko, L. & Hanan, N. P. On regreening and degradation in Sahelian watersheds. *Proc. Natl. Acad. Sci.* **112**, 12133–12138 (2015).
40. Brandt, M. *et al.* Ground- and satellite-based evidence of the biophysical mechanisms behind the greening Sahel. *Glob. Change Biol.* **21**, 1610–1620 (2015).
41. Dardel, C. *et al.* Re-greening Sahel: 30years of remote sensing data and field observations (Mali, Niger). *Remote Sens. Environ.* **140**, 350–364 (2014).
42. Fensholt, R. *et al.* Greenness in semi-arid areas across the globe 1981–2007 — an Earth Observing Satellite based analysis of trends and drivers. *Remote Sens. Environ.* **121**, 144–158 (2012).
43. Mbow, C., Brandt, M., Ouedraogo, I., de Leeuw, J. & Marshall, M. What Four Decades of Earth Observation Tell Us about Land Degradation in the Sahel? *Remote Sens.* **7**, 4048–4067 (2015).
44. Tian, F. *et al.* Remote sensing of vegetation dynamics in drylands: Evaluating vegetation optical depth (VOD) using AVHRR NDVI and in situ green biomass data over West African Sahel. *Remote Sens. Environ.* **177**, 265–276 (2016).
45. Trichon, V., Hiernaux, P., Walcker, R. & Mougin, E. The persistent decline of patterned woody vegetation: The tiger bush in the context of the regional Sahel greening trend. *Glob. Change Biol.* **24**, 2633–2648 (2018).

46. Christian, B. A., Dhinwa, P. S. & Ajai. Long term monitoring and assessment of desertification processes using medium & high resolution satellite data. *Appl. Geogr.* **97**, 10–24 (2018).
47. Jin, Q. & Wang, C. The greening of Northwest Indian subcontinent and reduction of dust abundance resulting from Indian summer monsoon revival. *Sci. Rep.* **8**, 4573 (2018).
48. Asoka, A. & Mishra, V. Prediction of vegetation anomalies to improve food security and water management in India. *Geophys. Res. Lett.* **42**, 5290–5298 (2015).
49. Bhanja, S. N. *et al.* Groundwater rejuvenation in parts of India influenced by water-policy change implementation. *Sci. Rep.* **7**, 7453 (2017).
50. Birkenholtz, T. Assessing India's drip-irrigation boom: efficiency, climate change and groundwater policy. *Water Int.* **42**, 663–677 (2017).
51. Rodell, M. *et al.* Emerging trends in global freshwater availability. *Nature* 1 (2018) doi:10.1038/s41586-018-0123-1.
52. Schwensow, N. I. *et al.* Rabbit haemorrhagic disease: virus persistence and adaptation in Australia. *Evol. Appl.* **7**, 1056–1067 (2014).
53. Bird, P., Mutze, G., Peacock, D. & Jennings, S. Damage caused by low-density exotic herbivore populations: the impact of introduced European rabbits on marsupial herbivores and Allocasuarina. *Biol. Invasions* **14**, 743–755 (2011).
54. Mutze, G., Bird, P., Cooke, B. & Henzell, R. Geographic and Seasonal Variation in the Impact of Rabbit Haemorrhagic Disease on European Rabbits, Oryctolagus cuniculus, and Rabbit Damage in Australia. in *Lagomorph Biology* (eds. Alves, P. D. P. C., Ferrand, P. D. N. & Hackländer, P. D. K.) 279–293 (Springer Berlin Heidelberg, 2008). doi:10.1007/978-3-540-72446-9_19.
55. Pedler, R. D. *et al.* Rabbit biocontrol and landscape-scale recovery of threatened desert mammals. *Conserv. Biol.* **30**, 774–782 (2016).
56. Cooke, B. D. & Mutze, G. J. How introduced rabbits Oryctolagus cuniculus limit the abundance of red kangaroos Macropus rufus and other native grazers in Australia. *Food Webs* **15**, e00079 (2018).
57. van Dijk, A. I. J. M. *et al.* The Millennium Drought in southeast Australia (2001–2009): Natural and human causes and implications for water resources, ecosystems, economy, and society. *Water Resour. Res.* **49**, 1040–1057 (2013).
58. Poulter, B. *et al.* Contribution of semi-arid ecosystems to interannual variability of the global carbon cycle. *Nature* **509**, 600–603 (2014).
59. Tong, X. *et al.* Increased vegetation growth and carbon stock in China karst via ecological engineering. *Nat. Sustain.* **1**, 44–50 (2018).
60. Fang, J. *et al.* Forest biomass carbon sinks in East Asia, with special reference to the relative contributions of forest expansion and forest growth. *Glob. Change Biol.* **20**, 2019–2030 (2014).
61. Webb, N. P. *et al.* Land degradation and climate change: building climate resilience in agriculture. *Front. Ecol. Environ.* **15**, 450–459 (2017).
62. UNWFP. Zambia | World Food Programme. *United Nations World Food Programme* http://www1.wfp.org/countries/zambia (2018).
