# Supplementary material for: Anthropogenic climate change has driven over 5 million km2 of drylands towards desertification
Source: Nat Commun. 2020 Jul 31;11:3853. doi: 10.1038/s41467-020-17710-7 (PMC7395722; doi:10.1038/s41467-020-17710-7)
Supplement: Supplementary file 2 — Peer Review File [file 41467_2020_17710_MOESM2_ESM.docx]

Peer Review Information

**Journal:** Nature Communications

**Manuscript title:** Click here to enter text.
Anthropogenic Climate change has driven over 5 million km2 of drylands towards desertification

**Corresponding author name(s):** Dr Arden Burrell

**Editorial notes:**

**Transferred manuscripts**

This manuscript has been previously reviewed at another journal that is not operating a transparent peer review scheme. This document only contains reviewer comments and rebuttal letters for versions considered at *Nature Communications*.

**Reviewer comments & decisions:**

**Reviewer comments, first version:**

Reviewer #1 (Remarks to the Author):

1. The authors’ revisions by adding new equations and text greatly improved the clarity of the CO2 attribution method. Many critical assumptions have now been pointed out. It, however, still deserves more discussion due to the simplicity of the method applied. I suggest two revisions for improvement. (1) Supplementary Figure 1 & 2 should be referenced in the methods text and discussed regarding C3 vs C4 pathways, as well as the spatial agreement/disagreement patterns shown in the two figures. (2) I suggest adding an additional figure to show the time series of annual, global mean values of NDVI(obs) and NDVI(adj), so that readers can at least visualize the temporal nature of this attribution.

2. Still, more clarity is needed for the comparison to previous results. At the very least, the authors should explicitly point out the spatial extents of (ecosystems included in) these studies and the differences it generated. If the main text does not have enough space, I would suggest adding a detailed comparison in the supplementary text.

3. I disagree combining “climate change” and “CO2 fertilization effect” into one conceptual category “anthropogenic climate change”. For the lack of a better term, “global environmental change” is more appropriate in this context, which is consistent with the term used by Zhu et al.


Reviewer #3 (Remarks to the Author):

The authors of the manuscript "Anthropogenic Climate change has driven over 5 million km2 of drylands towards desertification" attributed the vegetation greenness change of drylands to CO2, climate variability, climate change and land use based on the modified version of the TSS-RESTREND method. On combating the desertification, this study is meaningful and the subject could be of interest. However, I think there are several problems which the authors need to further explain or clarify.

Major comments:
1. The Intergovernmental Panel on Climate Change (2019) has already attributed the change of NDVI using the same method. The only difference of this study with IPCC is that the author subdivided climate to climate change and variability. I am worried that this study is not enough innovative comparing with previous studies.
Reference:
Intergovernmental Panel on Climate Change. Chapter 3: Desertification in the IPCC Special Report on Climate Change, Desertification, Land Degradation, Sustainable Land Management, Food Security, and Greenhouse gas fluxes in Terrestrial Ecosystems. (2019).

2. Please give the uncertain of method to estimate CO2 fertilization and spatial pattern of relative change of NDVI due to CO2 fertilization.

3. When authors verified their conclusion, they compared their result with previous studies. However, the direct proof was not given. I suggested authors give the evolution of different driver when they discussed main contribution to the local change of NDVI. For example, when they discussed the climate change, please give the evolution of local temperature and precipitation.

4. Line 93-96:Why climate change and land use could explain all change of NDVI after the role of CO2 fertilization removed? What about other factor, such as wildfire, nitrogen deposition, etc? For example, Zhu et al (2016) pointed that the contribution of nitrogen deposition is larger than climate change. Wildfire is now the prime factor next to climate change in some of the forest lands in the western United States, the Mediterranean area, and Australia (Neary, 2018).
Reference:
Zhu, Z. et al. Greening of the Earth and its drivers. Nat. Clim. Change 6, 791 (2016).
Neary, D. G. Wildfire contribution to desertification at local, regional, and global scales [Chapter 8]. In: Squires, Victor Roy; Ariapour, Ali, eds. Desertification: Desertification: Past, Current and Future Trends. Hauppauge, NY: Nova Science Publishers, Inc. p. 199-222 (2018).

5. In Figure 1c, the mean of other factor of change in NDVImax is approximately equal to the mean of climate change, the magnitude of other factor is more larger than that of climate variability and climate change. Why is there no region where other factor was the largest absolute driver in figure 1b and the total contribution of CO2 (44.1%), land use (28.2%), climate variability (14.6%) and climate change (13.1%) is 100%?

Other comments:
1. Line 147-150: In the Figure 4, I suggest you also give the changes in NDVImax attributed to other factor.
2. Line 367: subscript 2 ...CO2.
3. Line 395: There should be lowercase.
4. Line 429: subscript 2 .
5. Line 433-434: Keep the format consistent.
6. In order to keep the format of reference consistent, delete the 'doi' in Reference 5, 25, 27, 70, 72.

**Author rebuttal, first version:**

Reviewer #1 (Remarks to the Author):

1. The authors’ revisions by adding new equations and text greatly improved the clarity of the CO2 attribution method. Many critical assumptions have now been pointed out. It, however, still deserves more discussion due to the simplicity of the method applied. I suggest two revisions for improvement. (1) Supplementary Figure 1 & 2 should be referenced in the methods text and discussed regarding C3 vs C4 pathways, as well as the spatial agreement/disagreement patterns shown in the two figures. (2) I suggest adding an additional figure to show the time series of annual, global mean values of NDVI(obs) and NDVI(adj), so that readers can at least visualize the temporal nature of this attribution.

1) References to the supplementary materials added in methods:

*“.....The first ensemble assumes all plants are C3 and respond to elevated CO2 and the second ensemble assumes all plants are C4 with no eCO2 response (see Supplementary Figure 1 and Supplementary Figure 2).*

*However, recent surprising results from a long-term CO2 manipulation experiment in a dryland ecosystem have shown much higher responses in C4 plants*^1^*. For this reason, the results of both the C3 and C4 12-member ensembles are included in the supplementary material for those interested parties (Supplementary Figure 1 and Supplementary Figure 2).*

*As for the uncertainty in the approach we used to measure the CO2 fertilisation effect, the C3 and C4 12-member ensembles included in the supplementary material provide an estimate of the upper and lower bounds of CO2 responses (Supplementary Figure 1 and Supplementary Figure 2). ”*

Supplementary Figure 3 has been added which compares these ensembles further, and additional discussion has been added to Supplementary text 2.1 where the difference of between ensembles is discussed in relation to the impact of including CO2 on change attribution in specific regions.

*“	Our C3 ensemble, which ignores CO2 comes to the same conclusion. However, our mixed C3/C4 ensemble which includes CO2 finds that the CO2 is the dominant driver in this region (Figure 1b and Supplementary Figure 3c). Similarly, in the southern part of South America, our C3 ensemble which ignores CO2 finds Climate Change and*

*Climate Variability to be the dominant drivers, (Supplementary Figure 2 and Supplementary Figure 3c) which is consistent with Song et al.,*^2^*. However, when CO2 is included, large areas of negative land use become apparent (Figure 4d and Supplementary Figure 3a).”*

2) A figure showing the NDVIobs and NDVIadj has been added to supplementary material and the following has been added to the Methods:

A time series plot of the mean global NDVIobs and NDVIadj, is shown in Supplementary Figure 5 to highlight temporal nature of this attribution.

2. Still, more clarity is needed for the comparison to previous results. At the very least, the authors should explicitly point out the spatial extents of (ecosystems included in) these studies and the differences it generated. If the main text does not have enough space, I would suggest adding a detailed comparison in the supplementary text.

A detailed comparison has been included as Supplementary Discussion 2.

*“In the main part of this article we compare our results to two recent global studies by Song et al.,*^2^ *and Zhu et al.,*^3^ *which have examined the drivers of global vegetation change. Zhu et al.,*^3^ *used a 10 model ensemble with change attribution performed by running models with and without different drivers and then comparing the results. Song et al.,*^2^ *used AVHRR derived vegetation fractions to look at change in vegetation types, then performed attribution using high resolution imagery at 1500 locations across the globe. At each location, Song at al.,*^2^ *looked for “visible signs of human activity” and if these signs where present then the observed change was attributed to land use and if not then it was attributed to “indirect drivers such as climate change”.*

*These two studies highlight the existing inconsistency in the published literature, with the literature showing that the broad trends in vegetation change are consistent but there are large differences in attribution. The comparison between our study and these studies is not perfect due to different spatial domains. Our study only looks at the dryland biomes, with these making up only ~50% of land area that these global studies cover. Both global studies found that the importance of drivers varies between biomes. This section provides additional discussion and support for our argument that that the underlying assumptions made, and methods used in the attribution of change, are the source of much of the discrepancy between our findings and these previous studies.*

*2.1 The CO2 fertilisation effect*

*The impact of CO2 in drylands is well documented*^4^ *and as discussed in the main text, Song et al.,*^2^ *does not attribute change to CO2 which leads to Song et al., identifying Land Use and Climate as the dominate drivers of change. Supplementary Figure 2 shows a detailed breakdown of the results of our attribution approach if we assumed no CO2 fertilisation effect. An example of this can be seen in eastern China where Song et al.,*^2^ *finds land use driven change. Our C3 ensemble, which ignores CO2 comes to the same conclusion. However, our mixed C3/C4 ensemble which includes CO2 finds that the CO2 is the dominant driver in this region (Figure 1b and Supplementary Figure 3c). Similarly, in the southern part of South America, our C3 ensemble which ignores CO2 finds Climate Change and Climate Variability to be the dominant drivers, (Supplementary Figure 3c) which is consistent with Song et al.,*^2^*. However, when CO2 is included, large areas of negative land use become apparent (Figure 4d and Supplementary Figure 3a). Our conclusion that CO2 fertilisation is the dominate driver of vegetation change, is consistent with the findings of Zhu et al.,*^3^*. Figure 1b does not show areas where ‘Other Factors’ was dominant as this makes it more comparable to Song et al.,*^2^*. For interested parties, Supplementary Figure 3 shows the dominant driver in all three ensembles with Other Factors included which is more comparable to the result of Zhu et al.,*^3^*.*

*2.2 Land use*

*When comparing the attribution performed by Song et al.,*^2^ *to the Land Use changes detected by our approach (Figure 4d), there was generally broad agreement between large clusters of points. For example, in India and China where Song et al., found change was dominated by Land Use , we also found large land use driven changes, though in some places the changes attributed to Land Use were smaller in magnitude than the changes driven by CO2. This highlights the value of quantifying the impact and direction of all the drivers, not just the dominant one. Directly comparing our findings is not possible in all regions as the point-based attribution approach used by Song et al.,*^2^ *has*

*large gaps between attribution sites in places like Mongolia. In Mongolia there is strong evidence that grazing is the main Land Use impact* ^5^*, a result supported by our attribution approach which finds Land Use to be the dominant driver.*

*These findings differ considerably from the results of Zhu et al.,*^3^ *who found a significantly smaller Land Use effect. The models used by the Zhu et al., study do not calculate the land use changes but instead rely on prescribed datasets to incorporate processes like deforestation* ^3,6–10^*^.^ Existing work has shown that models are very sensitive to the land use data used to force them* ^11,12^*^.^ The data used to drive the TRENDY models used by Zhu et al., (the HYDE dataset) is national data downscaled to the model’s grid and does not include many important processes like grazing which is dominant land use in many dryland region’s* ^3,7,8^*^.^ An example of this issue can be seen when looking at Mongolia where grazing of goats is known to be driving widespread change* ^5^*. In this region we find large areas where Land Use is the dominate driver of change, while Zhu et al., attributes change in the region to Other Factors. Furthermore, the way the TRENDY models implement Land Use varies significantly which leads the estimates to “differ significantly in magnitude, and sometimes also in sign”*^3^*. The tendency for modelling approaches to underestimate Land Use has been documented by several recent studies^22,23^.*

*2.3 Climate change and variability*

*By definition, dryland areas are very sensitive to water availability and are predominantly found in areas with high natural climate variability*^13^*. Climate Change and Climate Variability is another area where our findings differ significantly from the results of Zhu et al.,*^3^*. The TRENDY models are not able to measure climate variability and the estimate of climate change impacts vary considerably between models*^3^*. A good example of the difference between our findings and that of Zhu et al.,*^3^ *can be seen in the Sahel where Zhu et al., attribute much of the change in vegetation to Climate Change, while our findings, as well as previous work in that region, attribute it to decadal climate variability (see Supplementary Text 1.1). The approach used by Song et al.,*^2^ *does not distinguish between climate change and climate variability. Previous studies have raised serious concerns about the validity of trend detection and attribution in dryland vegetation, especially in short vegetation like grasses, that is performed without properly accounting for natural climate variability*^13–15^*.*

*2.4 Nitrogen deposition*

*Nitrogen deposition is driver of vegetation change that our approach was not able to quantify but was included in the Zhu et al.,*^3^*analysis. Zhu et al.,*^3^ *found nitrogen deposition to be the second largest driver globally, but noted that this finding was uncertain given that only two of their models could be run with and without the nitrogen deposition. There is good evidence that nitrogen deposition is not an important driver of vegetation change in drylands. A recent meta-analysis on the impacts of nitrogen deposition on plant species found that drylands are not sensitive to increased N loads because they are overwhelmingly water limited and, with the exception of a small part of the southwestern United States, “are mainly present in regions with very low N deposition”* ^16^ *This conclusion is supported by the findings of Zhu et al.,*^3^ *who found that Nitrogen played little to no role in dryland areas.*

*2.5 Fires*

*The impact of fire on long-term dryland vegetation change is not quantified by our method or by either of the global studies undertaken by Song et al., and Zhu et al. Dryland fires are an important contributing factor to the carbon cycle and on atmospheric aerosols* ^17^*. The natural fire return interval in drylands is very short, with many dryland tree species having evolved adaptations, such as epicormic resprouting (the rapid reestablishment of extensive leaf area) to survive and recover after fires*^18^*. The grasses and shrubs are also adapted to recover rapidly after fires. This rapid recovery is not apparent in all ecosystems, with growing evidence that the dry forests in the alpine parts of the southwestern United States are experiencing fire-driven desertification* ^19^*. Similarly, when developing the TSS-RESTREND method used in this study, Burrell et al.,* ^15^ *found that while fire did not seem to impact long term vegetation trends or change the ecosystem structure in most locations, in the southeastern alpine zone extreme fire events had caused permanent forest loss. In this case, TSS-RESTREND detects the change and attributes it to Land Use.”*

3. I disagree combining “climate change” and “CO2 fertilization effect” into one conceptual category

“anthropogenic climate change”. For the lack of a better term, “global environmental change” is more appropriate in this context, which is consistent with the term used by Zhu et al.

The key anthropogenic change discussed here is the increase in atmospheric CO2 concentration. This, in turn, has a direct impact on the climate, causing it to change. It is not possible to have anthropogenic climate change that is not caused (mostly) by increases in CO2. These two things are aspects of the same anthropogenic cause, hence we have reason to discuss them together (they are also discussed separately). We realise that this is not common in the literature and hence we clearly define our meaning in the second sentence of the manuscript “anthropogenic climate change, which includes both changes in water availability driven by trends in precipitation and increases in temperature ^10,11^ , as well as increased water use efficiency (carbon gain per unit of water lost) in response to rising atmospheric CO2 ^12^”, “Global Environmental Change” is a much broader term that would also encompass land use changes (amongst other things) that we are explicitly excluding from “anthropogenic climate change”2,20–23.

Reviewer #3 (Remarks to the Author):

The authors of the manuscript "Anthropogenic Climate change has driven over 5 million km2 of drylands towards desertification" attributed the vegetation greenness change of drylands to CO2, climate variability, climate change and land use based on the modified version of the TSS-RESTREND method. On combating the desertification, this study is meaningful and the subject could be of interest. However, I think there are several problems which the authors need to further explain or clarify.

Major comments:

1. The Intergovernmental Panel on Climate Change (2019) has already attributed the change of NDVI using the same method. The only difference of this study with IPCC is that the author subdivided climate to climate change and variability. I am worried that this study is not enough innovative comparing with previous studies. Reference:

Intergovernmental Panel on Climate Change. Chapter 3: Desertification in the IPCC Special Report on Climate Change, Desertification, Land Degradation, Sustainable Land Management, Food Security, and Greenhouse gas fluxes in Terrestrial Ecosystems. (2019).

The IPCC report includes a figure based on an earlier version of TSS-RESTREND but does not include analysis presented here. Additional discussion that highlights the difference has been added to the methods:

*“TSS-RESTREND differs from existing dryland trend attribution methods in that it is able to capture both the long term trends and the step changes in NDVI that occur in regions where ecosystems have experienced significant structural changes*^15^*. To do this TSS-RESTREND incorporates a phenological change detection method*^24^ *to identify structural changes in the ecosystem which manifest as breakpoints in the NDVI time series. We used TSS-RESTREND v2.15, which has been updated to use both precipitation and temperature ^25^, to calculate the Vegetation Climate Relationship (VCR) using the per pixel optimal precipitation and temperature accumulation periods*^15,25^*. TSS-RESTREND was applied to the NDVIadj, with the Land Use (LU) driven component calculated using an ordinary least squared regression between the residuals of the VCR and time, accounting for any detected structural changes to the ecosystem. A similar approach was presented by the IPCC*^13^*, though that approach does not separate climate change from climate variability and also assumes that all dryland plants follow a C3 photosynthetic pathway.”*

2. Please give the uncertain of method to estimate CO2 fertilization and spatial pattern of relative change of NDVI due to CO2 fertilization.

Additional explanation has been added to the methods to make that clear.
*“Determining statistical significance*

*For both Obs trend and CO2 driven change in NDVI, the Spearman's rank correlation co-efficient test was used to measure statistical significance for each pixel* ^26^*. In order to determine field significance and account for the multiple testing problem the Benjamini–Hochberg procedure was then applied to these p-values to control the False Discovery Rate (FDR) (αFDR = 0.10)*^27^*. As for the uncertainty in the approach we used to measure the CO2 fertilisation effect, the C3 and C4 12-member ensembles included in the supplementary material provide an estimate of the upper and lower bounds of CO2 responses (Supplementary Figure 1 and Supplementary Figure 2). “*

3. When authors verified their conclusion, they compared their result with previous studies. However, the direct proof was not given. I suggested authors give the evolution of different driver when they discussed main contribution to the local change of NDVI. For example, when they discussed the climate change, please give the evolution of local temperature and precipitation.

We are uncertain exactly what the review meant by this comment and which section they are referring too. However, we have made a number of changes that we hope addresses this concern:

1. We added a regional breakdown of the dominate drivers (Supplementary Figure 4)
2. We add time-series of the observed, climate change driven and climate variability driven mean regional precipitation and temperature anomalies (Supplementary Figure 8 and 9)

We added references to these anomalies in the text:

*“	Similarly, the negative impacts of decreased rainfall over the semi-arid Caatinga forest of Brazil has*

*amplified the effects of widespread deforestation and grazing intensification. The decrease in rainfall in South America results from both climate change (mean precipitation anomaly 1982-2015 ≈ -0.2) as well as a negative phase of climate variability (mean precipitation anomaly ≤ 0) from 2009 to 2014 (Supplementary Figure 8). For further discussion of these regions and comparison to regional studies see Supplementary Discussion 1.1..............”*

In the Supplementary material:
1.

*“In this section we discuss our findings compared to regional studies. When discussing the impact of climate change we report the regional means of the accumulated precipitation anomaly (APA) and accumulated temperature anomaly (ATA) driven by climate change and climate variability. The calculation of these anomalies is described in the methods and a full regional time-series is included in Supplementary Figure 8 and Supplementary Figure 9*.

1.1 a:	*These negative drivers have been compounded by a long-term climate change driven increase in*

*temperature (mean ATACC ≈ 1 from 1982 to 2015) resulting in the so called ‘Aral Sea disaster’* ^28^

1.1 b:	*In this region, widespread deforestation and grazing intensification has compounded the effect of	a long*

*term decline in precipitation* ^29–33^ *driven by climate change (mean APACC ≈ -0.2 from 1982 to 2015) which has led*

*to widespread desertification* ^34^

1.2 a:......*Our attribution of greening over the Sahel, is consistent with the conclusion that the greening is driven by increased rainfall caused by the wet phase of the natural decadal variability reinforced by the CO2 effect* 35–41*. The shift from the dry phase to the wet phase of climate variability can be seen in Supplementary Figure 8. Between 1982 and 1988 the Sahel had below average rainfall (mean APAcv ≤ 0) including 1984 which had the largest negative precipitation anomaly observed in any region over the period 1982 to 2015 (APAcv(1984) ≈ -1.1). In contrast, the only year from the period 2010 to 2015 with below average precipitation was 2011 with and APACV ≈ -0.1.*

1.2 c:	*While a lack of large scale field observations makes this hard to confirm, there is evidence for the*

*recovery of both vegetation and native animal populations* ^42–45^*^.^ The evidence for the impact of climate variability is well documented in Australia. From 2001 to 2009 much of Australia experienced the Millennium Drought*^46^ *which was characterised by below average rainfall and above average temperatures (Supplementary Figure 8 and Supplementary Figure 9). The drought came to an end in 2010-2011 with extreme precipitation associated with a strong La Nina event which is the largest positive precipitation anomaly observed in any region over the period 1982 to 2015 (APAcv ≈ 1.1). This rainfall caused a massive increase in vegetation productivity with Australia accounting for 60% of the global land carbon sink that year*^47^*.* “

We also added a section to the methods explaining how these anomaly values were calculated;

*“When discussing regional drivers of vegetation change in the main text as well as in Supplementary Discussion 1, we report the mean climate anomaly rather than the accumulated precipitation and temperature values to allow comparison of different accumulation and offset periods of different pixels. For the observed accumulated precipitation and temperature, the anomaly was calculated on a per pixel basis using:*

*(3)*	*=* − *.*

*.*

*Where z=anomaly, n is the year, x=observed value, μ=mean of the per pixel accumulated precipitation or temperature, σ = standard deviation of the per pixel accumulated precipitation or temperature. The temperature and precipitation anomaly attributed to climate change was calculated using:*

*× (* − 0*)*

*(4)*

*=	.*

*Where β is the per pixel trend in accumulated precipitation or temperature, n0 is the first year of the analysis (1982). The temperature and precipitation anomaly attributed to climate variability was calculated using:*

| (5) = | () − .  . |
| --- | --- |

*Where x(adj) is the detrended precipitation or temperature. Regional breakdowns of the time-series of observed, climate change and climate variability driven precipitation and temperature anomaly are shown in Supplementary Figure 8 and Supplementary Figure 9 respectively*

4. Line 93-96: Why climate change and land use could explain all change of NDVI after the role of CO2 fertilization removed? What about other factor, such as wildfire, nitrogen deposition, etc? For example, Zhu et al (2016) pointed that the contribution of nitrogen deposition is larger than climate change. Wildfire is now the prime factor next to climate change in some of the forest lands in the western United States, the Mediterranean area, and Australia (Neary, 2018).

Reference:

Zhu, Z. et al. Greening of the Earth and its drivers. Nat. Clim. Change 6, 791 (2016).

Neary, D. G. Wildfire contribution to desertification at local, regional, and global scales [Chapter 8]. In: Squires, Victor Roy; Ariapour, Ali, eds. Desertification: Desertification: Past, Current and Future Trends. Hauppauge, NY: Nova Science Publishers, Inc. p. 199-222 (2018).

Other Factors was not included here because we were comparing our results to Song et al., who did not include other factors. As such we compared dominate attributed factors. We have added considerable extra explanation of this as Supplementary Discussion 2.

*“	Figure 1b does not show areas where ‘Other Factors’ was dominant to be comparable to Song et al.,***^2^***. For interested parties, Supplementary Figure 3 shows the dominant driver in all three ensembles with Other Factors included which is more comparable to the result of Zhu et al.,***^3^***.”*

**Supplementary Discussion 2 also includes a specific discussion of Fires and Nitrogen deposition.** *“2.4 Nitrogen deposition*

*Nitrogen deposition is driver of vegetation change that our approach was not able to quantify but was included in the Zhu et al.,***^3^***analysis. Zhu et al.,***^3^** *found nitrogen deposition to be the second largest driver globally, but noted that this finding was uncertain given that only two of their models could be run with and without the nitrogen deposition. There is good evidence that nitrogen deposition is not an important driver of vegetation change in drylands. A recent meta-analysis on the impacts of nitrogen deposition on plant species found that drylands are not sensitive to increased N loads because they are overwhelmingly water limited and, with the exception of a small part of the southwestern United States, “are mainly present in regions with very low N deposition”* **^16^** *This conclusion is supported by the findings of Zhu et al.,***^3^** *who found that Nitrogen played little to no role in dryland areas.*

*2.5 Fires*

*The impact of fire on long-term dryland vegetation change is not quantified by our method or by either of the global studies undertaken by Song et al., and Zhu et al. Dryland fires are an important contributing factor to the carbon cycle and on atmospheric aerosols* **^17^***. The natural fire return interval in drylands is very short, with many dryland tree species having evolved adaptations, such as epicormic resprouting (the rapid reestablishment of extensive leaf area) to survive and recover after fires***^18^***. The grasses and shrubs are also adapted to recover rapidly after fires. This rapid recovery is not apparent in all ecosystems, with growing evidence that the dry forests in the alpine parts of the southwestern United States are experiencing fire-driven desertification* **^19^***. Similarly, when developing the TSS-RESTREND method used in this study, Burrell et al.,* **^15^** *found that while fire did not seem to impact long term vegetation trends or change the ecosystem structure in most locations, in the southeastern alpine zone extreme fire events had caused permanent forest loss. In this case, TSS-RESTREND detects the change and attributes it to Land Use. “*

5. In Figure 1c, the mean of other factor of change in NDVImax is approximately equal to the mean of climate change, the magnitude of other factor is more larger than that of climate variability and climate change. Why is there no region where other factor was the largest absolute driver in figure 1b and the total contribution of CO2 (44.1%), land use (28.2%), climate variability (14.6%) and climate change (13.1%) is 100%?

The focus of this section was comparing our findings to two different existing studies. The method used by Song et al., do not explicitly consider the contribution of other factors and in order to facilitate comparison we only showed the attributed factors in that figure. A version of the figure that includes other factors has been added to Supplementary Figure 3. We have added extra explanation of this in Supplementary Discussion 2 and have added a reference to that discussion in the main text

Main

*“For comparison, we repeated our ensemble analysis, removing the role of CO2 fertilisation (Supplementary Figure 2) and found that 60.4% of global dryland vegetation change would have been attributed to land use and 39.6% to climate change and variability combined which is consistent despite a difference in the study domains and the attribution methods (See Supplementary Discussion 2).”*

**Supplementary Discussion 2**

*“	Figure 1b does not show areas where ‘Other Factors’ was dominant to be comparable to Song et al.,***^2^***. For interested parties, Supplementary Figure 3 shows the dominant driver in all three ensembles with Other Factors included which is more comparable to the result of Zhu et al.,***^3^***.”*

Other comments:

1. Line 147-150: In the Figure 4, I suggest you also give the changes in NDVImax attributed to other factor.

This is already included in Supplementary Material 7. Figure 4 is focused on mapping the attributable fraction with Other Factors being the unattributable component.

**The following are all changes to the references of the manuscript:**

2. Line 367: subscript 2 ...CO2.
   Done
3. Line 395: There should be lowercase.
   Done
4. Line 429: subscript 2 .
   Done
5. Line 433-434: Keep the format consistent.
   Done
6. In order to keep the format of reference consistent, delete the 'doi' in Reference 5, 25, 27, 70, 72. Done

**References used in this document**

1. Reich, P. B., Hobbie, S. E., Lee, T. D. & Pastore, M. A. Unexpected reversal of C3 versus C4 grass response to elevated CO2 during a 20-year field experiment. *Science* **360**, 317–320 (2018).
2. Song, X.-P. *et al.* Global land change from 1982 to 2016. *Nature* 1 (2018) doi:10.1038/s41586-018-0411-9.
3. Zhu, Z. *et al.* Greening of the Earth and its drivers. *Nature Climate Change* **6**, 791 (2016).
4. Ukkola, A. M. *et al.* Reduced streamflow in water-stressed climates consistent with CO2 effects on vegetation. *Nature Clim. Change* **6**, 75–78 (2016).
5. Liu, Y. Y. *et al.* Changing Climate and Overgrazing Are Decimating Mongolian Steppes. *PLoS ONE* **8**, e57599 (2013).
6. Calle, L. *et al.* Regional carbon fluxes from land use and land cover change in Asia, 1980–2009. *Environ. Res. Lett.* **11**, 074011 (2016).
7. Li, W. *et al.* Land-use and land-cover change carbon emissions between 1901 and 2012 constrained by biomass observations. *Biogeosciences* **14**, 5053–5067 (2017).
8. Peng, S. *et al.* Sensitivity of land use change emission estimates to historical land use and land cover mapping: Land Use and Land Cover Mapping. *Global Biogeochemical Cycles* **31**, 626–643 (2017).
9. Tharammal, T., Bala, G., Devaraju, N. & Nemani, R. A review of the major drivers of the terrestrial carbon uptake: model-based assessments, consensus, and uncertainties. *Environ. Res. Lett.* **14**, 093005 (2019).
10. Yue, C. *et al.* Representing anthropogenic gross land use change, wood harvest, and forest age dynamics in a global vegetation model ORCHIDEE-MICT v8.4.2. *Geosci. Model Dev.* **11**, 409–428 (2018).
11. Arneth, A. *et al.* Historical carbon dioxide emissions caused by land-use changes are possibly larger than assumed. *Nature Geoscience* **10**, 79–84 (2017).
12. Bayer, A. D. *et al.* Uncertainties in the land-use flux resulting from land-use change reconstructions and gross land transitions. *Earth Syst. Dynam.* **8**, 91–111 (2017).
13. Intergovernmental Panel on Climate Change (IPCC). *Chapter 3: Desertification in the IPCC Special Report on Climate Change, Desertification, Land Degradation, Sustainable Land Management, Food Security, and Greenhouse gas fluxes in Terrestrial Ecosystems.* <https://www.ipcc.ch/site/assets/uploads/2019/08/2d.-Chapter-3_FINAL.pdf> (2019).
14. Wessels, K. J., van den Bergh, F. & Scholes, R. J. Limits to detectability of land degradation by trend analysis of vegetation index data. *Remote Sensing of Environment* **125**, 10–22 (2012).
15. Burrell, A. L., Evans, J. P. & Liu, Y. Detecting dryland degradation using Time Series Segmentation and Residual Trend analysis (TSS-RESTREND). *Remote Sensing of Environment* **197**, 43–57 (2017).
16. Bobbink, R. *et al.* Global assessment of nitrogen deposition effects on terrestrial plant diversity: a synthesis. *Ecological Applications* **20**, 30–59 (2010).
17. Rogers, B. M., Balch, J. K., Goetz, S. J., Lehmann, C. E. R. & Turetsky, M. Focus on changing fire regimes: interactions with climate, ecosystems, and society. *Environ. Res. Lett.* **15**, 030201 (2020).
18. Burrows, G. E. Buds, bushfires and resprouting in the eucalypts. *Aust. J. Bot.* **61**, 331–349 (2013).
19. Neary, D. G. Wildfire contribution to desertification at local, regional, and global scales [Chapter 8]. *In: Squires, Victor Roy; Ariapour, Ali, eds. Desertification: Desertification: Past, Current and Future Trends. Hauppauge, NY: Nova Science Publishers, Inc. p. 199-222.* 199–222 (2018).
20. Borer, E. T., Grace, J. B., Harpole, W. S., MacDougall, A. S. & Seabloom, E. W. A decade of insights into grassland ecosystem responses to global environmental change. *Nature Ecology & Evolution* **1**, 1–7 (2017).
21. Pyhälä, A. *et al.* Global environmental change: local perceptions, understandings, and explanations. *Ecol Soc* **21**, (2016).
22. Lang, P. L. M., Willems, F. M., Scheepens, J. F., Burbano, H. A. & Bossdorf, O. Using herbaria to study global environmental change. *New Phytologist* **221**, 110–122 (2019).
23. Franklin, J., Serra-Diaz, J. M., Syphard, A. D. & Regan, H. M. Global change and terrestrial plant community dynamics. *PNAS* **113**, 3725–3734 (2016).
24. Verbesselt, J., Hyndman, R., Zeileis, A. & Culvenor, D. Phenological change detection while accounting for abrupt and gradual trends in satellite image time series. *Remote Sensing of Environment* **114**, 2970–2980 (2010).
25. Burrell, A. L., Evans, J. P. & Liu, Y. The Addition of Temperature to the TSS-RESTREND Methodology Significantly Improves the Detection of Dryland Degradation. *IEEE Journal of Selected Topics in Applied Earth Observations and Remote Sensing* 1–7 (2019) doi:10.1109/JSTARS.2019.2906466.
26. Yue, S., Pilon, P. & Cavadias, G. Power of the Mann–Kendall and Spearman’s rho tests for detecting monotonic trends in hydrological series. *Journal of Hydrology* **259**, 254–271 (2002).
27. Wilks, D. S. “The Stippling Shows Statistically Significant Grid Points”: How Research Results are Routinely Overstated and Overinterpreted, and What to Do about It. *Bull. Amer. Meteor. Soc.* **97**, 2263–2273 (2016).
28. Lioubimtseva, E. A multi-scale assessment of human vulnerability to climate change in the Aral Sea basin. *Environ Earth Sci* **73**, 719–729 (2015).
29. Marinho, F. P., Mazzochini, G. G., Manhães, A. P., Weisser, W. W. & Ganade, G. Effects of past and present land use on vegetation cover and regeneration in a tropical dryland forest. *Journal of Arid Environments* **132**, 26–33 (2016).
30. Novaes, R. L. M., Felix, S. & Souza, R. de F. Brazil: Save Caatinga from drought disaster. *Nature* <https://www.nature.com/articles/498170a> (2013) doi:10.1038/498170a.
31. Ribeiro, E. M. S., Arroyo-Rodríguez, V., Santos, B. A., Tabarelli, M. & Leal, I. R. Chronic anthropogenic disturbance drives the biological impoverishment of the Brazilian Caatinga vegetation. *Journal of Applied Ecology* **52**, 611–620 (2015).
32. Mariano, D. A. *et al.* Use of remote sensing indicators to assess effects of drought and human-induced land degradation on ecosystem health in Northeastern Brazil. *Remote Sensing of Environment* **213**, 129–143 (2018).
33. Lausier, A. M. & Jain, S. Overlooked Trends in Observed Global Annual Precipitation Reveal Underestimated Risks. *Sci Rep* **8**, 1–7 (2018).
34. Tomasella, J. *et al.* Desertification trends in the Northeast of Brazil over the period 2000–2016. *International Journal of Applied Earth Observation and Geoinformation* **73**, 197–206 (2018).
35. Brandt, M. *et al.* Ground- and satellite-based evidence of the biophysical mechanisms behind the greening Sahel. *Global Change Biology* **21**, 1610–1620 (2015).
36. Dardel, C. *et al.* Re-greening Sahel: 30years of remote sensing data and field observations (Mali, Niger). *Remote Sensing of Environment* **140**, 350–364 (2014).
37. Fensholt, R. *et al.* Greenness in semi-arid areas across the globe 1981–2007 — an Earth Observing Satellite based analysis of trends and drivers. *Remote Sensing of Environment* **121**, 144–158 (2012).
38. Kaptué, A. T., Prihodko, L. & Hanan, N. P. On regreening and degradation in Sahelian watersheds. *PNAS* **112**, 12133–12138 (2015).
39. Mbow, C., Brandt, M., Ouedraogo, I., de Leeuw, J. & Marshall, M. What Four Decades of Earth Observation Tell Us about Land Degradation in the Sahel? *Remote Sensing* **7**, 4048–4067 (2015).
40. Tian, F. *et al.* Remote sensing of vegetation dynamics in drylands: Evaluating vegetation optical depth (VOD) using AVHRR NDVI and in situ green biomass data over West African Sahel. *Remote Sensing of Environment* **177**, 265–276 (2016).
41. Trichon, V., Hiernaux, P., Walcker, R. & Mougin, E. The persistent decline of patterned woody vegetation: The tiger bush in the context of the regional Sahel greening trend. *Global Change Biology* **24**, 2633–2648 (2018).
42. Bird, P., Mutze, G., Peacock, D. & Jennings, S. Damage caused by low-density exotic herbivore populations: the impact of introduced European rabbits on marsupial herbivores and Allocasuarina. *Biol Invasions* **14**, 743–755 (2011).
43. Mutze, G., Bird, P., Cooke, B. & Henzell, R. Geographic and Seasonal Variation in the Impact of Rabbit Haemorrhagic Disease on European Rabbits, Oryctolagus cuniculus, and Rabbit Damage in Australia. in *Lagomorph Biology* (eds. Alves, P. D. P. C., Ferrand, P. D. N. & Hackländer, P. D. K.) 279–293 (Springer Berlin Heidelberg, 2008). doi:10.1007/978-3-540-72446-9_19.
44. Pedler, R. D. *et al.* Rabbit biocontrol and landscape-scale recovery of threatened desert mammals. *Conservation Biology* **30**, 774–782 (2016).
45. Cooke, B. D. & Mutze, G. J. How introduced rabbits Oryctolagus cuniculus limit the abundance of red kangaroos Macropus rufus and other native grazers in Australia. *Food Webs* **15**, e00079 (2018).
46. van Dijk, A. I. J. M. *et al.* The Millennium Drought in southeast Australia (2001–2009): Natural and human causes and implications for water resources, ecosystems, economy, and society. *Water Resour. Res.* **49**, 1040–1057 (2013).
47. Poulter, B. *et al.* Contribution of semi-arid ecosystems to interannual variability of the global carbon cycle. *Nature* **509**, 600–603 (2014).

**Reviewer comments, second version:**

Reviewer #1 (Remarks to the Author):

I reserve my previous comment#3 that combing “climate change” and “CO2 fertilization effect” into one category is not appropriate. The reason is that although CO2 contributes the most radiative forcing, climate change is caused by CO2 as well as other important anthropogenic greenhouse gases, e.g. methane, which does not have the same impact on vegetation as CO2. However, this is a minor comment compared to all other analyses presented in the paper. Apart from this, my other comments have been sufficiently addressed in the revised manuscript and I do not have any more comments. Publication is recommended.


Reviewer #3 (Remarks to the Author):

The current revised manuscript has improved in the contents. I suggest to publish the manuscript in the present form except for a minor correction:

Line 12-13: When talking about the global land vulnerability to anthropogenic climate change and human activities, such as land use change, the authors should cite other reference, e.g.
Huang J, Zhang G, Zhang Y, Guan X,Wei Y, Guo R. Global desertification vulnerability to climatechange and human activities.Land Degrad Dev. 2020;1–12.
Reynolds, J. F., Smith, D. M. S., Lambin, E. F., Turner, B., Mortimore, M., Batterbury, S. P., & Herrick, J. E.. Global desertification: building a science for dryland development. Science, 2007, 316(5826), 847-851.

Lind 380-382: Actually, Turkmenistan, Kazakhstan and Uzbekistan are located in Central Asia.

**Author rebuttal, second version:**

**Reviewer #1 (Remarks to the Author):**

**I reserve my previous comment#3 that combing "climate change" and "CO2 fertilization effect" into one category is not appropriate. The reason is that although CO2 contributes the most radiative forcing, climate change is caused by CO2 as well as other important anthropogenic greenhouse gases, e.g. methane, which does not have the same impact on vegetation as CO2. However, this is a minor comment compared to all other analyses presented in the paper. Apart from this, my other comments have been sufficiently addressed in the revised manuscript and I do not have any more comments. Publication is recommended.**

**Addition information has been added in the methods to further clarify our use of Anthropogenic Climate Change and to address the reviewers concerns:**

*"Calculating the impact of Anthropogenic Climate Change*

**In this manuscript we use the term Anthropogenic Climate Change to refer to both changes in water availability driven by trends in precipitation and increases in temperature^14,23^, as well as increased water use efficiency (carbon gain per unit of water lost) in response to rising atmospheric CO2^8,15^. The impact of Anthropogenic Climate Change (ACC) is calculated using** *ACC = CO2 + CC***. While we acknowledge that this considering CO2 fertilisation and Climate Change together is not common in the literature, these two things are aspects of the same anthropogenic cause, hence we have reason to discuss them together. It should be noted that while the methodology used in this study is able to capture the effects of other anthropogenic greenhouse gases like methane in the climate change component, we cannot separately quantify any direct impact that changes in the amount of these gasses will have on vegetation."**

**Reviewer #3 (Remarks to the Author):**

**The current revised manuscript has improved in the contents. I suggest to publish the manuscript in the present form except for a minor correction:**

**Line 12-13: When talking about the global land vulnerability to anthropogenic climate change and human activities, such as land use change, the authors should cite other reference, e.g.**

**Huang J, Zhang G, Zhang Y, Guan X,Wei Y, Guo R. Global desertification vulnerability to climatechange and human activities.Land Degrad Dev. 2020;1-12.**

**Reynolds, J. F., Smith, D. M. S., Lambin, E. F., Turner, B., Mortimore, M., Batterbury, S. P., & Herrick, J. E.. Global desertification: building a science for dryland development. Science, 2007, 316(5826), 847-851.**

**All references where removed from the abstract to fit with Nature Communications style. Both of the reviewer's references have been added to the body of the main text. They are Reference 4 and Reference 22 in the main manuscript.**

**Lind 380-382: Actually, Turkmenistan, Kazakhstan and Uzbekistan are located in Central Asia. Section change to** *Central and Western Asia*

**Reviewer comments, third version:**

**Author rebuttal, third version:**
